# Endocytic pathway inhibition attenuates extracellular vesicle-induced reduction of chemosensitivity to bortezomib in multiple myeloma cells

**DOI:** 10.7150/thno.47996

**Published:** 2021-01-01

**Authors:** Chenggong Tu, Zhimin Du, Hui Zhang, Yueyuan Feng, Yujun Qi, Yongjiang Zheng, Jinbao Liu, Jinheng Wang

**Affiliations:** 1 Affiliated Cancer Hospital & Institute of Guangzhou Medical University, Guangzhou 510095, China; 2 Guangzhou Municipal and Guangdong Provincial Key Laboratory of Protein Modification and Degradation; State Key Laboratory of Respiratory Disease; School of Basic Medical Sciences, Guangzhou Medical University, Guangzhou 511436, China; 3 School of Nursing, Guangzhou Medical University, Guangzhou 510182, China; 4 Zhengzhou University School of Medicine, Zhengzhou 450052, China; 5 Department of Hematology, The Third Affiliated Hospital of Sun Yat-Sen University, Guangzhou 510630, China

**Keywords:** extracellular vesicles, exosomes, multiple myeloma, endocytosis, chemosensitivity

## Abstract

Extracellular vesicles (EVs), including exosomes and microvesicles, derived from bone marrow stromal cells (BMSCs) have been demonstrated as key factors in the progression and drug resistance of multiple myeloma (MM). EV uptake involves a variety of mechanisms which largely depend on the vesicle origin and recipient cell type. The aim of the present study was to identify the mechanisms involved in the uptake of BMSC-derived small EVs (sEVs) by MM cells, and to evaluate the anti-MM effect of targeting this process.

**Methods:** Human BMSC-derived sEVs were identified by transmission electron microscopy, nanoparticle tracking analysis, and western blot. The effects of chemical inhibitors and shRNA-mediated knockdown of endocytosis-associated genes on sEV uptake and cell apoptosis were analyzed by flow cytometry. The anti-MM effect of blocking sEV uptake was evaluated *in vitro* and in a xenograft MM mouse model.

**Results:** sEVs derived from BMSC were taken up by MM cells in a time- and dose-dependent manner, and subsequently promoted MM cell cycling and reduced their chemosensitivity to bortezomib. Chemical endocytosis inhibitors targeting heparin sulphate proteoglycans, actin, tyrosine kinase, dynamin-2, sodium/proton exchangers, or phosphoinositide 3-kinases significantly reduced MM cell internalization of BMSC-derived sEVs. Moreover, shRNA-mediated knockdown of endocytosis-associated proteins, including caveolin-1, flotillin-1, clathrin heavy chain, and dynamin-2 in MM cells suppressed sEV uptake. Furthermore, an endocytosis inhibitor targeting dynamin-2 preferentially suppressed the uptake of sEV by primary MM cells *ex vivo* and enhanced the anti-MM effects of bortezomib *in vitro* and in a mouse model.

**Conclusion:** Clathrin- and caveolin-dependent endocytosis and macropinocytosis are the predominant routes of sEV-mediated communication between BMSCs and MM cells, and inhibiting endocytosis attenuates sEV-induced reduction of chemosensitivity to bortezomib, and thus enhances its anti-MM properties.

## Introduction

Extracellular vesicles (EVs), including exosomes and microvesicles, are small membranous particles released by parent cells. EVs mediate the local and distant cell-to-cell communication by delivering their cargo into recipient cells 1. The EV-mediated transfer of a range of molecules, including proteins, lipids, mRNAs and miRNAs, significantly alters the recipient cell phenotype 2-4. An increasing number of studies have demonstrated that by targeting stromal cells 5, 6, immune cells 7, 8, and vascular cells^8^, EVs derived from either normal or tumor cells regulate the maintenance and modification of the BM microenvironment. As a consequence, angiogenesis 9, cytokine secretion 5, inflammation 10, as well as tumor cell proliferation, invasiveness, and metastasis 11, 12, are modulated by EV internalization. For these functional effects to occur, EVs must first be taken up by target cells via numerous molecular mechanisms, including membrane fusion, clathrin- or caveolin-dependent endocytosis, phagocytosis, and macropinocytosis 13, 14. The routes of uptake depends on the expression of specific surface proteins on both the vesicle and the recipient cell, and thus a cell is capable of EV internalization via a number of different mechanisms 13, 15. Dissection of the biological mechanisms underlying EV internalization by specific types of recipient cell will facilitate the improved understanding of EV-mediated communication, and the development of novel strategies to target this process.

Multiple myeloma (MM), a hematological malignancy localized in the bone marrow (BM), is characterized by the malignant proliferation and accumulation of monoclonal plasma cells 16-18. BM is composed of hematopoietic and non-hematopoietic cells, extracellular matrix and a liquid compartment 19. The interaction between MM and BM-derived cells, which is primarily mediated by direct cell-to-cell contact, soluble factors and EVs, is pivotal for MM progression 20-23. Among the BM-derived cells, BM stromal cells (BMSCs; also known as mesenchymal stromal cells) have been reported to directly promote MM cell growth, survival and drug resistance through cell-to-cell contact and the secretion of various cytokines, chemokines and growth factors 24. Moreover, these stromal cells also communicate with other BM-derived cells, such as dendritic, natural killer and myeloid-derived suppressor cells (MDSCs), to modify the BM microenvironment and thus indirectly influence MM progression 25, 26. Ours and other previous studies have reported that BMSC-derived small EVs (sEVs, 30-200 nm) are involved in the proliferation and drug resistance of MM cells 27, 28 and that they are able to indirectly facilitate MM progression by promoting MDSC activation 7. Considering the crucial role of BMSC-derived sEVs in MM progression, it is important to fully elucidate the cellular mechanisms involved in sEV internalization by MM cells, and thus develop effective strategies to inhibit sEV-mediated communication.

Using chemical inhibitors to target different endocytic pathways, as well as shRNA-mediated knockdown of endocytosis-associated genes, we sought to characterize the cellular mechanisms by which MM cells internalize human BMSC-derived sEVs. In addition, we also evaluated the anti-MM effect of blocking sEV uptake both *in vitro* and using a MM mouse model.

## Materials and Methods

### Regents and antibodies

Bortezomib and endocytosis inhibitors, including heparin, chlorpromazine, amiloride, dynasore, wortmannin, omeprazole, and genistein were purchased from Selleck Chemicals (Houston, TX, USA). 5-(N-Ethyl-Nisopropyl) amiloride (EIPA) was obtained from Cayman Chemical (Ann Arbor, MI, USA). Antibodies against flotillin-1 (Flot1, D2V7J, 18634T, 1:1000), glyceraldehyde-3-phosphate dehydrogenase (GAPDH, D16H11, 5174S, 1:1000), calreticulin (D3E6, 12238T, 1:1000), caveolin-1 (CAV-1, D46G3, 3267T, 1:1000), and clathrin heavy chain (CLTC, D3C6, 4796S, 1:1000) were purchased from (Cell Signaling Technology, Danvers, MA, USA). Antibodies against dynamin-2 (DNM2, EPR9053, ab151555, 1:1000) and CD9 (EPR2949, ab92726, 1:2000) were bought from Abcam (Cambridge, United Kingdom). Anti-human CD63 antibody (Ts63, 10628D, 1:250) was obtained from Thermo Fisher Scientific (Waltham, MA, USA). IRDye 680RD or 800CW goat anti-mouse/rabbit IgG secondary antibodies (1:10000) were purchased from LI-COR Biosciences (Lincoln, NE, USA).

### Cell culture

Human MM cell lines, including H929, U266, MM1S, and RPMI 8226, were purchased from China Center for Type Culture Collection (Wuhan, China) and cultured in RPMI 1640 medium (Invitrogen, Carlsbad, CA, USA) supplemented with 10% fetal bovine serum (FBS, Biological Industries, Beit HaEmek, Israel), 2mM L-glutamine (Biological Industries), and 100 U/mL penicillin/streptomycin (Biological Industries). BM samples were obtained from newly diagnosed MM patients after patients' informed consent and all research that involves human samples was approved by Ethical Committee for Clinical Medicine Research of The Third Affiliated Hospital of Sun Yat-Sen University. The clinical information of MM patients are listed in Table S1. BM mononuclear cells (BMMCs) were isolated from these BM samples via a Lymphoprep (Stemcell Technologies, Inc., BC, Canada) gradient. BMMCs were cryopreserved in 90% FBS and 10% DMSO for long-term storage in liquid nitrogen or cultured in OriCell Human MSC Culture medium (Cyagen Biosciences, Inc., CA, USA) at 37 °C as described previously 14. BMSCs were then obtained after removing non-adherent cells and continuously cultured in OriCell Human MSC Culture medium. These BMSCs were used within 10 passages.

### Isolation and quantification of sEV

Human BMSCs were washed with phosphate buffer saline (PBS) once and cultured in serum-free DMEM medium (Invitrogen) for 24 h. sEVs were isolated from these conditioned medium as described previously 8, 27. Briefly, the conditioned medium was filtered using 0.22 μm pore filters (Millipore, Germany) and concentrated using Ultra-15 Centrifugal Filter Units (100KD, Millipore). These concentrated conditioned medium was washed with 10 ml PBS twice to further reduce possible contamination from proteins. After filtering using 0.22 μm filter, these medium was incubated with ExoQuick-TC exosome precipitation solution (System Biosciences, CA, USA) at 4 °C overnight. sEVs were collected by centrifugation and resuspended in PBS. The concentration of sEV proteins was determined using a Pierce BCA Protein Assay Kit (Thermo Fisher Scientific). 20 mL conditioned medium were collected from 2×10^6^ BMSCs and about 0.8 μg sEVs were obtained from 1 mL conditioned medium. We have submitted all relevant data of our experiments to the EV-TRACK knowledgebase (EV-TRACK ID: EV200118) 29. EV METRIC score of our entry is 75% and the average EV METRIC score of 179 submitted experiments in 2020 is 49%.

### Transmission electron microscopy (TEM) and nanoparticle tracking analysis

The concentration of isolated sEVs was adjusted to 1 μg/mL in PBS. 10 μL sEVs suspension was dropped on a formvar-carbon coated grid and incubated at room temperature for 20 min. Grids were dried and stained with 1% uranyl acetate (Zhongjingkeyi Technology, Beijing, China) for another 10min.The excess liquid was removed with a filter paper. The grid was dried at room temperature for 30 min and the sEVs on the grid were observed using a JEM-1400 Plus transmission electron microscope (JEOL, Tokyo, Japan) at 120 kV. Size distribution of isolated sEVs was examined via a Zetaview Nanoparticle Tracking Analyzer (Particle Metrix GmbH, Meerbusch, Germany). Normally, ~1.2×10^8^ particles were detected in 1 μg sEVs.

### Western blot analysis

BMSCs or MM cells were lysed in radioimmunoprecipitation assay buffer containing proteinase inhibitor cocktail on ice for 10 min. Lysates of cells was centrifuged at 12,000g for 5 min and the supernatant was collected. The protein concentration in cell lysates or sEV suspension were quantified using the Pierce BCA Protein Assay Kit. Cell lysates or sEV suspension was mixed with loading buffer and heated at 95℃ for 5 min. These samples were loaded onto polyacrylamide gel containing sodium dodecyl sulfate and subjected to electrophoresis. The proteins in the gel were transferred to polyvinylidene fluoride membranes and the membrane was blocked with blocking buffer for 1 h at room temperature. After incubation with primary antibodies and florescent dye-labeled secondary antibodies, the protein bands on the membrane were visualized and obtained using an Odyssey CLx imaging system (LI-COR Biosciences).

### Fluorescent labeling of sEV

BMSC-derived sEVs were incubated with a lipophilic carbocyanine fluorescent dye DID (20 nM, Invitrogen) at 37 °C for 30 min. Unincorporated DID was removed using Exosome Spin Columns (Life Technologies) according to the manufacturer's instruction. DID in PBS without sEVs were processed using Exosome Spin Columns in the same way to obtain a DID control solution.

### sEV uptake assay

DID-labeled sEVs were added into H929, MM1S, RPMI8226, or U266 MM cells (3×10^5^ cells/mL) and after 6, 12, 24, or 48 h of culture, the cells were collected and diluted with PBS for fluorescence detection using flow cytometry. MM cells pre-treated with or without endocytosis inhibitors for 30 min were incubated with 50 μg/mL DID-labeled sEVs for another 4 h and the cells were collected. The fluorescent signal in these cells were determined using a BD Accuri C6 flow cytometer (Becton Dickinson and Company, Franklin Lakes, NJ, USA).

### Cell cycle analysis

H929, MM1S, RPMI8226, or U266 cells (3×10^5^ cells/mL) cultured in serum-free RPMI 1640 medium were treated with or without DID-labeled sEVs derived from BMSCs for 48 h. Cells were collected, washed once with PBS, and incubated with propidium iodide (50 µg/mL, Beyotime Biotechnology, Shanghai, China) solution containing 0.1% Triton X-100 (Sigma-Aldrich, St. Louis, MO, USA), 1 mg/mL citrate sodium (Kermel Chemical, Tianjin, China), and 100 µg/mL RNaseA (Beyotime Biotechnology). PI and DID signals in MM cells were measured using the BD Accuri C6 flow cytometer and the cell cycle was analyzed using FlowJo software (FlowJo LLC, Ashland, OR, USA).

### Cell viability and apoptosis assays

In the absence or presence of bortezomib (7.5 nM for MM1S, RPMI8226, and U266 cells, 30 nM for H929 cells), MM cells (3×10^5^ cells/mL) were treated with or without BMSC-derived sEVs for 48 h and the viability was measured with the Cell Titer glo Luminescent Viability assay (Promega, Madison, WI, USA). MM cells were treated with or without endocytosis inhibitors for 4.5 h and the cell viability was determined using the Cell Titer glo Luminescent Viability assay. All viability assays were performed in triplicate and repeated in three independent experiments. For apoptosis analysis, the treated cells were stained with Annexin V-FITC (Biolegend, San Diego, CA, USA) and 7-Aminoactinomycin D (7-AAD, Biolegend) and apoptotic cells were determined using a CytoFLEX S flow cytometer (Beckman Coulter, Brea, CA, USA).

### Lentivirus Transfections and uptake assays

Lentivirus expressing shRNAs against endocytosis-related genes, including CAV-1, CLTC, DNM2, and Flot1, were purchased from GenePharma (Shanghai, China). RPMI8226 and U266 cells were seeded at a density of 4×10^5^/well in 24 well plates and incubated with lentivirus expressing shRNAs or negative control shRNAs. After 48h, cells were treated with 1 µg/mL puromycin (Beyotime Biotechnology) and expanded in the presence of puromycin for another 14 days. These cells were subjected to either western blot analysis for assessing the knockdown efficiency or sEV uptake assay.

### sEV uptake in primary BM cells

Thawed BMMCs (1×10^6^ cells/mL) in RPMI 1640 medium supplemented with 10% FBS were pre-treated with or without dynasore at different final concentrations (12.5, 25, or 50 µM) for 30 min and then incubated with 50 μg/mL DID-labeled sEVs for another 4 h. These cells were collected and stained with anti-mouse CD45-PE (Biolegend, 304007) and anti-CD38-Pacific Blue (Biolegend, 356627) for 30 min at room temperature (RT) and then stained with Annexin V-FITC in binding buffer (Biolegend) for 15 min at RT. After staining, fluorescence intensities were measured using a CytoFLEX S flow cytometer and analyzed using Flowjo software.

### In vivo study

Female NOD-Prkdc^scid^ IL2rg^tm1^/Bcgen (B-NDG) mice at 4 weeks of ages were purchased from Biocytogen (Wakefield, MA, USA) and housed and treated following conditions approved by the Institutional Animal Care and Use Committee of Guangzhou Medical University. A total of 3×10^6^ U266 cells in 200 μl PBS expressing GFP and luciferase (U266-GFP-Luc) were intravenously injected into mice to generate a human myeloma xenograft mouse model. Mice bearing U266-GFP-Luc cells were randomized into three groups prior to vehicle or drug treatment. Intraperitoneal injection of 0.3 or 0.6 mg/kg of bortezomib and/or 10 or 100 mg/kg of dynasore in 200 μl PBS was used for in vivo treatment. For bioluminescence imaging, mice were intraperitoneally injected with 150 mg/kg of D-luciferin in 200 μl PBS (PerkinElmer, Waltham, MA, USA) and bioluminescence signal was captured using a IVIS Lumina III in vivo imaging System (PerkinElmer). Bioluminescence intensity was quantified using a Living Images software (PerkinElmer). For the Kaplan-Meier curve, moribund mice showing hind limb paraplegia were considered as the 'endpoint' and sacrificed.

### Statistical analysis

The Shapiro-Wilk test was used to test whether the data are normally distributed. To compare two groups, Student's t-test was used for the normal distribution data and Mann-Whitney U test was used for non-normally distributed data. For comparing multiple groups, one-way ANOVA followed by multiple compressions was used. Statistical significance was determined using GraphPad Prism 7.0 software (GraphPad Software, La Jolla, CA, USA). *P* < 0.05 was regarded as statistically significant.

## Results

### Human BMSC-derived sEVs are internalized by MM cells

**Figure 1:**
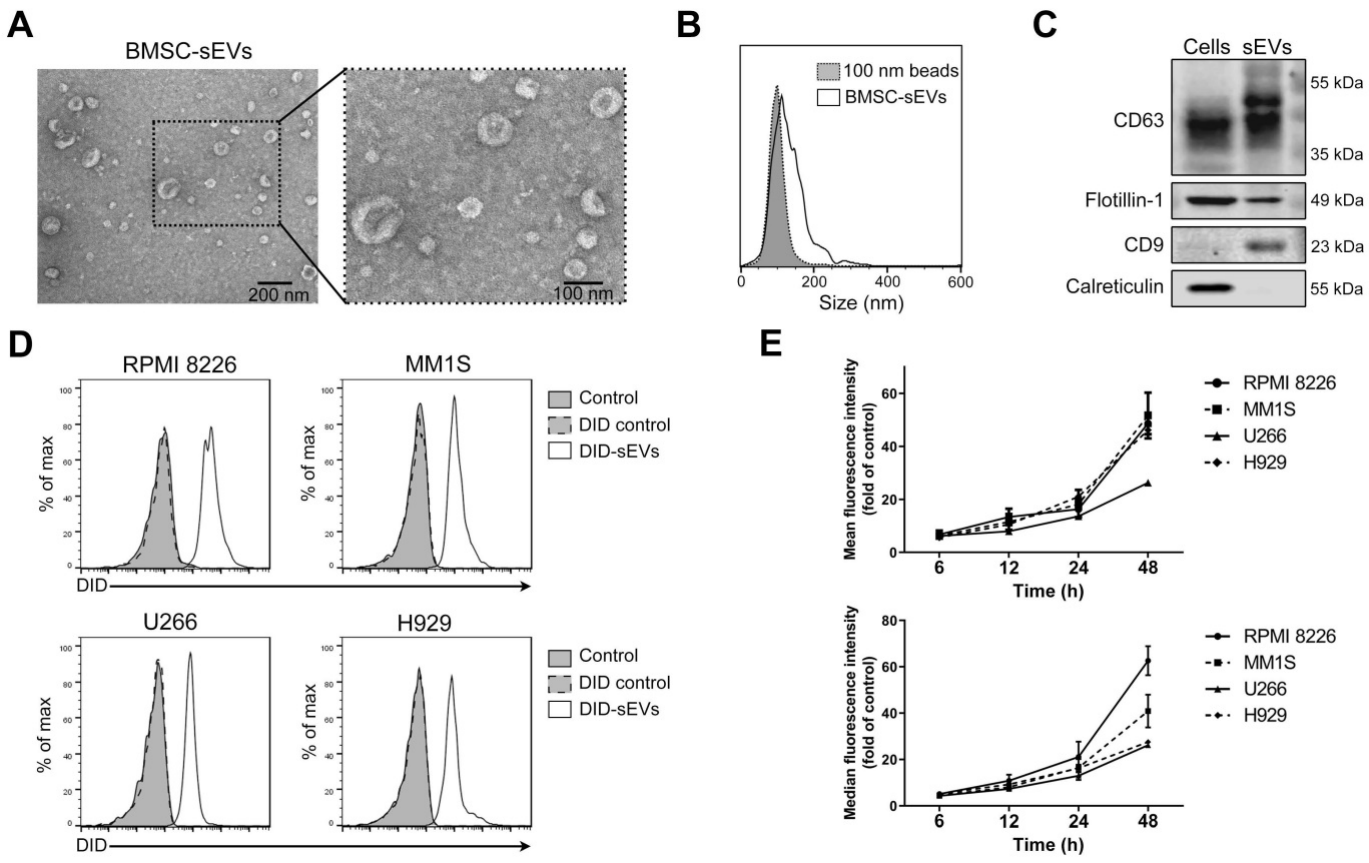
** Human BMSC-derived sEVs can be taken by MM cells.** (A) Transmission electron microscopy images of human BMSC-derived sEVs. (B) Size distribution of human BMSC-derived sEVs was determined using the nanoparticle tracking analysis. (C) Exosomal positive markers, including CD63, CD9, flotillin-1, as well as a negative marker calreticulin, in BMSC and BMSC-derived sEV lysate were measured using western blot. (D) 50 μg/mL DID-labeled BMSC-derived sEVs or DID control solution was added to four MM cell lines, including RPMI 8226, MM1S, U266, and H929. After 24 h of culture, the fluorescence signal of DID in these cells was examined using flow cytometry. PBS was added to cells and included as a control. (E) 50 μg/mL DID-labeled BMSC-derived sEVs were added to four MM cell lines and the mean and median fluorescence intensity of DID in these cells was determined using flow cytometry after the culture for indicated times. n=3. Error bar, mean ± SD.

Firstly, BMSCs isolated from a MM patient were cultured with serum-free medium for 24 h and the conditioned medium was collected for sEV isolation. BMSC viability was measured at that time and only very few (< 1%) cells were apoptotic (Figure S1A). sEVs were then isolated from these conditioned medium and their morphology and size were confirmed using TEM. Typical cup-shaped membrane particles with a diameter of 50 to 120 nm were observed on the TEM images (Figure 1A). Nanoparticle tracking analysis was used to assess sEV size distribution, showing a size distribution of 50 to 200 nm (Figure 1B). The expression of various exosomal markers, including CD63, flotillin-1 (Flot1), and CD9, was detected in these sEVs, and calreticulin, a protein not expressed by sEVs, was not detected (Figure 1C). To confirm the uptake of BMSC-derived sEVs by MM cells, four different MM cell lines, including RPMI 8226, MM1S, U266, and H929, were cultured with sEVs fluorescently labeled with DID. After 24 h of culture, almost all MM cells exhibited DID fluorescence, indicating sEV internalization at this time point (Figure 1D). The fluorescence intensities of these cells were then determined at additional time points, and a time-dependent increase in fluorescent intensity was revealed in all four cell lines (Figure 1E). After long-term incubation, the fluorescence signal in MM cells continued to increase until 4-5 days later when it began to decrease (Figure S1B). In addition, sEVs were taken up by MM cells in a dose-dependent manner (Figure S1C).

### BMSC-derived sEVs promote MM cell cycling

**Figure 2:**
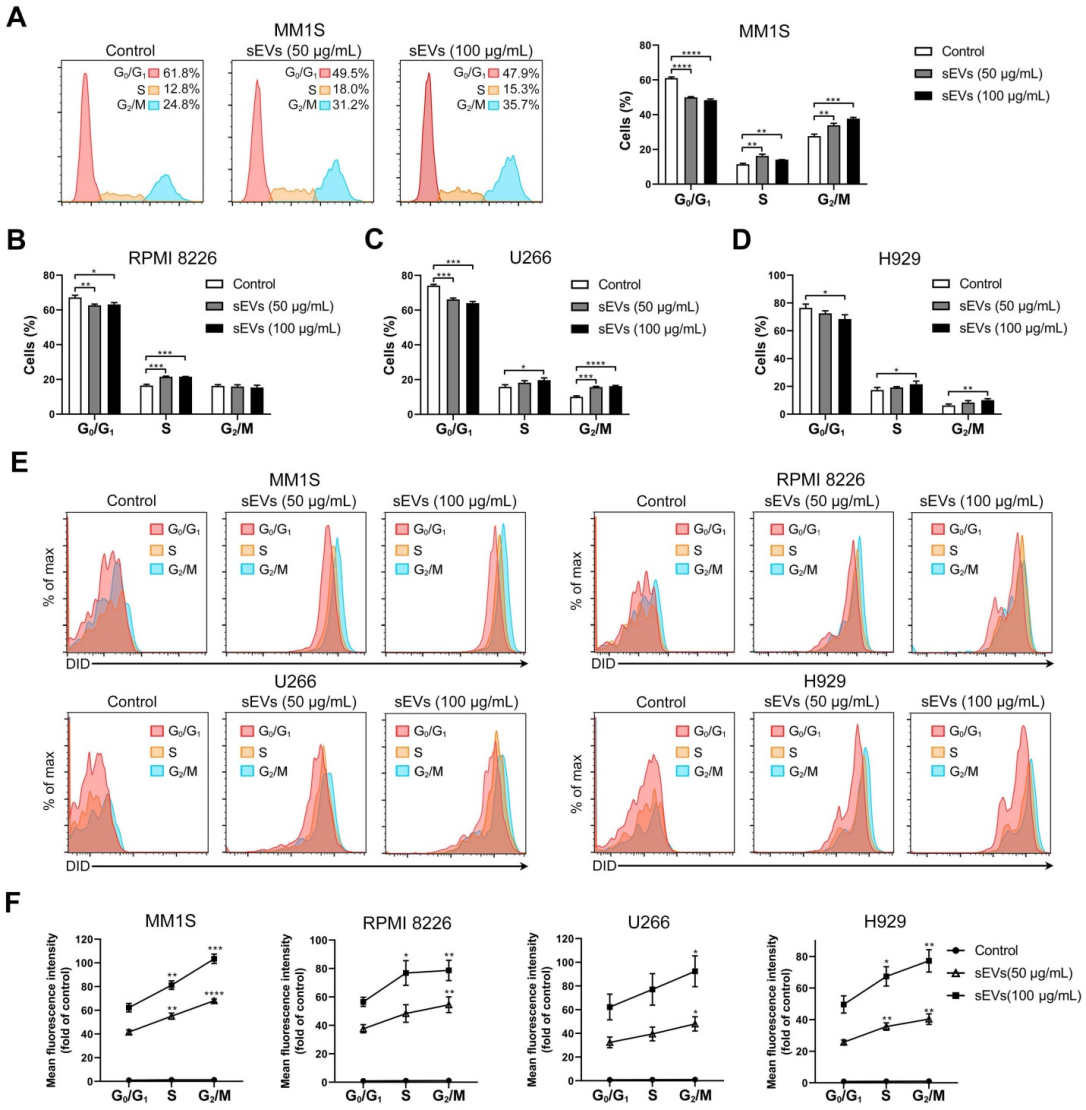
** Human BMSC-derived sEVs promote MM cell cycle.** (A) MM1S cells were cultured with or without 50 or 100 μg/mL BMSC-derived sEVs for 48 h and the cell cycle was determined using PI staining and flow cytometry. Representative flow cytometry plots are shown in the left panel. The proportions of MM1S cells in different cell cycle stages were measured using flow cytometry and displayed using histogram (right panel). (B) RPMI 8226, (C) U266, or (D) H929 cells were cultured with or without 50 or 100 μg/mL BMSC-derived sEVs for 48 h and the proportions of cells in different cell cycle phases were determined using PI staining and flow cytometry. One representative result in triplicate of three experiments was presented by histograms. Similar results were obtained in three independent experiments. (E) MM1S, RPMI 8226, U266, or H929 cells were cultured with or without 50 or 100 μg/mL DID-labeled BMSC-derived sEVs for 48 h and representative flow cytometry plots are shown. (F) Mean fluorescence intensity of DID in the cells at different cell cycle stages were determined using PI staining and flow cytometry and presented by histograms. One-way ANOVA followed by multiple compressions was used for comparing multiple groups. Error bar, mean ± SD. *P < 0.05, **P < 0.01, ***P < 0.001, ****P < 0.0001.

Next, BMSC-derived sEVs were added to MM1S MM cells and cell cycle analysis was conducted. sEV internalization increased the percentages of cells in the S and G_2_/M phases and decreased the number of cells in the G_0_/G_1_ phase (Figure 2A). Similar results were observed in U266 and H929 MM cells (Figure 2C-D and S2). In RPMI 8226 cells, sEV internalization increased the proportion of cells in the S phase and decreased those in the G_0_/G_1_ phase, but did not alter the percentage of cells in the G_2_/M phase (Figure 2B and S2). As sEVs were found to influence cell cycle progression, the degree of sEV internalization was determined at different cell cycle phases. DID-labeled sEVs were added to the MM cells and fluorescence intensity was assessed at different phases. The highest signal was detected in cells in the G_2_/M phase, and the lowest fluorescence intensity was observed in cells in the G_0_/G_1_ phase, which predominantly represents cells in the interphase state (Figure 2E-F). Similar results were observed in all four cell lines, indicating that dividing cells internalize more sEVs readily than those in a resting state.

### BMSC-derived sEVs attenuate MM cell chemosensitivity to bortezomib

**Figure 3:**
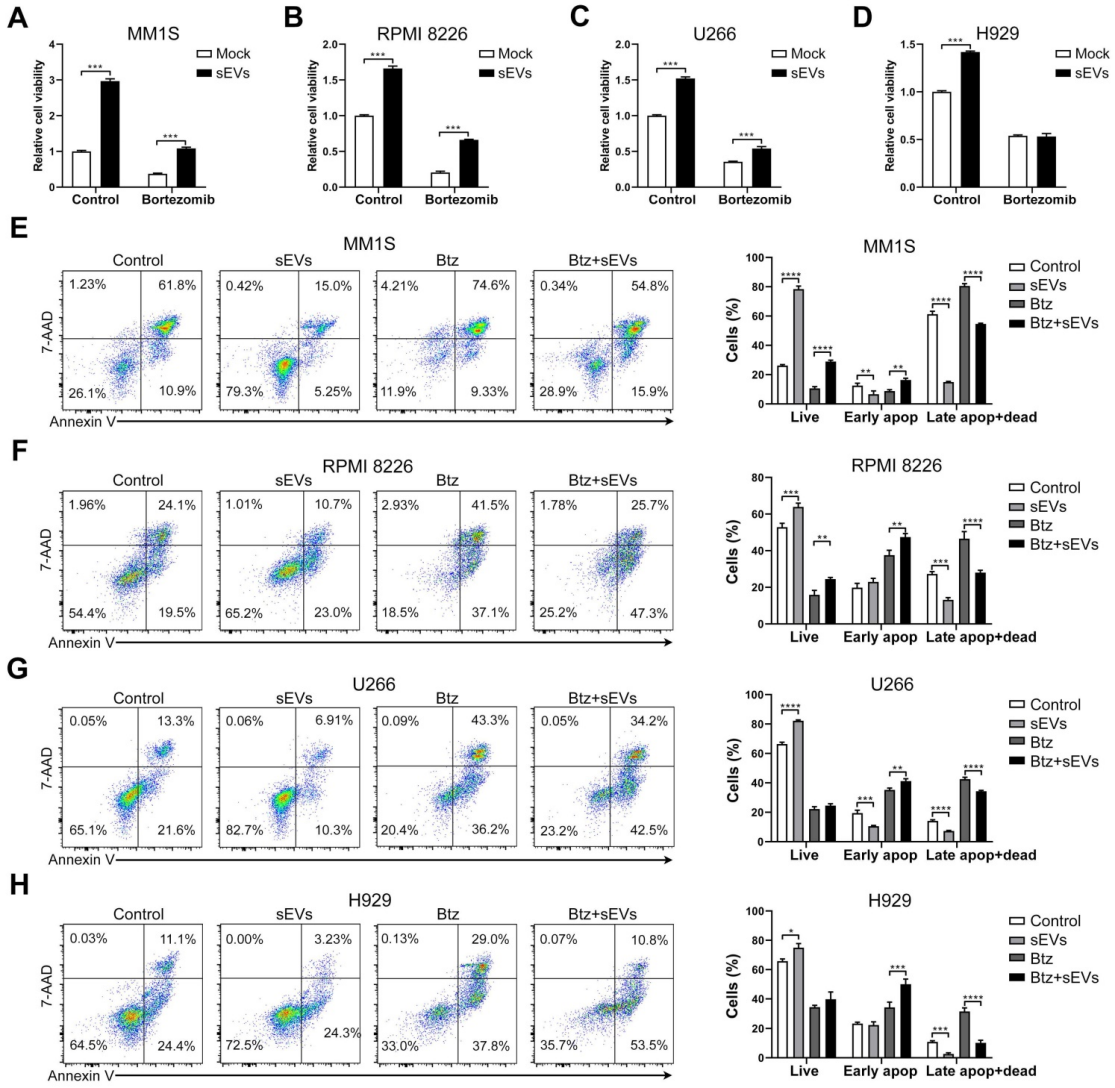
** Human BMSC-derived sEVs facilitate the drug resistance of MM cells to bortezomib.** (A) MM1S, (B) RPMI 8226, (C) U266, or (D) H929 cells were cultured with or without 100 μg/mL BMSC-derived sEVs in the absence or presence of bortezomib (7.5 nM for MM1S, RPMI 8226, and U266 cells, 30 nM for H929 cells) for 48 h and cell viability was measured. One representative result in triplicate of three experiments was presented by histograms. Similar results were obtained in three independent experiments. Student's t-test was used for comparing two groups. (E) MM1S, (F) RPMI 8226, (G) U266, or (H) H929 cells were treated with or without 100 μg/mL BMSC-derived sEVs in the absence or presence of bortezomib for 48 h. Apoptotic cells were determined using 7-AAD and Annexin-V staining and flow cytometry. The proportions of live, early apoptotic or late apoptotic and dead cells were analyzed and presented by histograms. One-way ANOVA followed by multiple compressions was used for comparing multiple groups. Error bar, mean ± SD. *P < 0.05, **P < 0.01, ***P < 0.001, ****P < 0.0001.

Previous studies have shown that BMSC-derived sEVs facilitated the growth of MM cells 27, 28, and we confirmed that these sEVs were able to dose-dependently increase the viability of RPMI 8226 and U266 MM cells (Figure S3A). Time-dependent promotion was only observed in U266 cells (Figure S3A). In addition, the supernatant obtained from sEVs suspension has a negligible effect on MM cell viability (Figure S3B). Next, MM cells were cultured with BMSC-derived sEVs and their chemosensitivity to bortezomib was evaluated. In three of the MM cell lines (MM1S, RPMI8226, and U266), the total cell viability was increased by sEV internalization regardless of bortezomib treatment (Figure 3A-C and S3C), compared with those without sEV administration. This finding suggests that sEV internalization reduces the chemosensitivity to bortezomib. In H929 cells, sEV internalization increased their cell viability only in the absence of bortezomib (Figure 3D and S3C). In MM1S and RPMI 8226 cells treated with bortezomib, sEV administration significantly increased the proportion of live and early apoptotic cells and decreased the percentage of late apoptotic and dead cells (Figure 3E-F). Although sEV administration did not increase the proportion of live U266 and H929 cells following treatment with bortezomib, a significant increase in early apoptotic cells was observed, in addition to a decrease in dead and late apoptotic and cells (Figure 3G-H). These findings indicate that BMSC-derived sEVs markedly delay bortezomib-induced death in MM cells, and thus attenuate their sensitivity to bortezomib.

### Endocytosis inhibitors suppress MM cell sEV internalization

**Figure 4:**
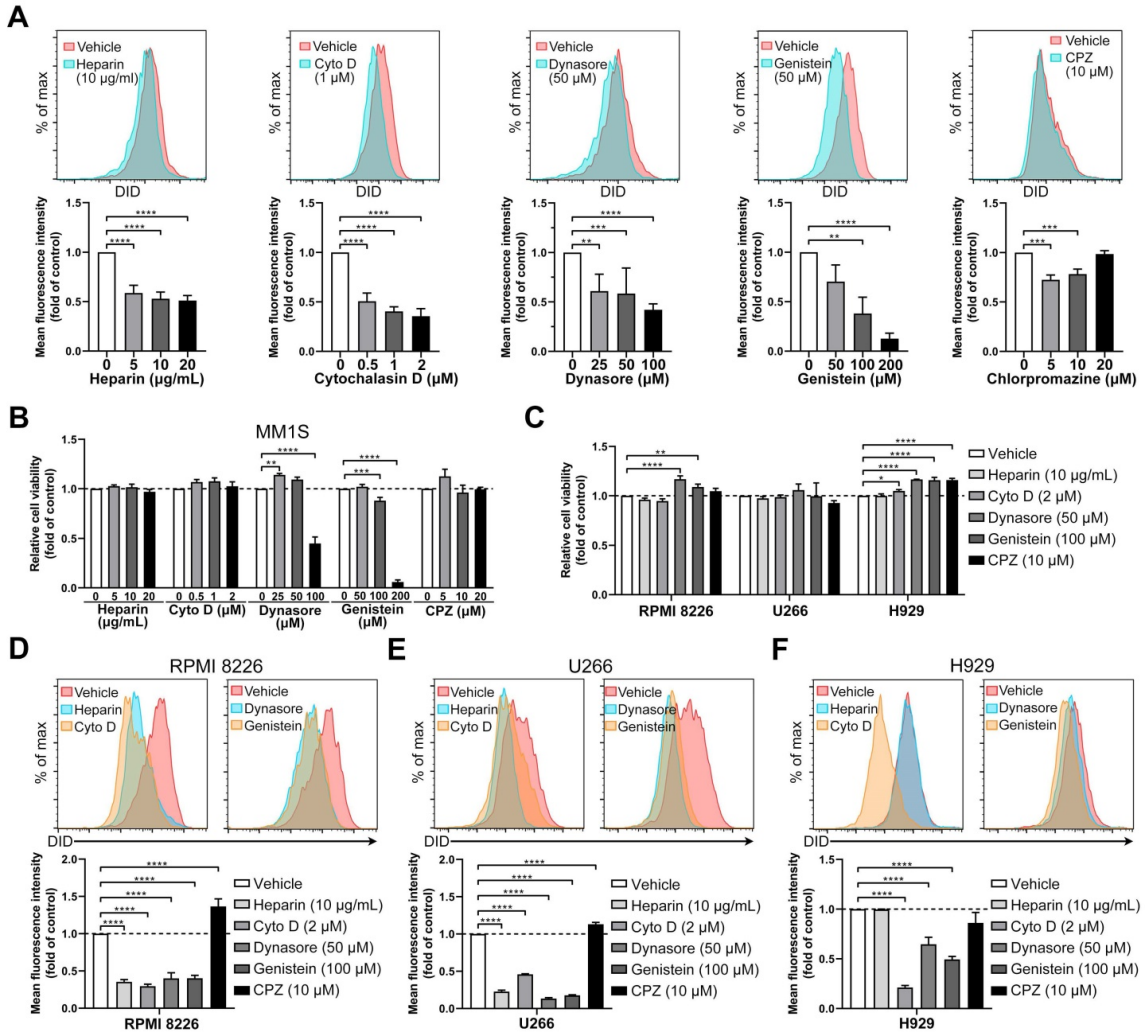
** Endocytosis inhibitors decrease the uptake of BMSC-derived sEVs by MM cells.** (A) MM1S cells pre-treated with heparin, cytochalasin D (Cyto D), dynasore, genistein, or chlorpromazine (CPZ) at the indicated concentrations for 30 min were cultured with 50 μg/mL DID-labeled BMSC-derived sEVs for another 4 h. The mean fluorescence intensity of DID in these cells was determined using flow cytometry. (B) MM1S cells were treated with heparin, cytochalasin D, dynasore, genistein, or chlorpromazine at the indicated concentrations for 4.5 h in the absence of sEVs and their cell viability was measured. (C) RPMI 8226, U266, or H929 cells were treated with heparin, Cyto D, dynasore, genistein, or CPZ at the indicated concentration for 4.5 h in the absence of sEVs and their cell viability was measured. (D) RPMI 8226, (E) U266, or (F) H929 cells were pre-treated with heparin, Cyto D, dynasore, genistein, or CPZ at the indicated concentration for 30 min and cultured with 50 μg/mL DID-labeled BMSC-derived sEVs for another 4 h. The mean fluorescence intensity of DID in these cells was determined using flow cytometry. One-way ANOVA followed by multiple compressions was used for comparing multiple groups. n=3. Error bar, mean ± SD. *P < 0.05, **P < 0.01, ***P < 0.001, ****P < 0.0001.

sEVs and exosomes can be taken up by recipient cells via multiple different mechanisms, including clathrin- or caveolin- mediated endocytosis, macropinocytosis, phagocytosis, lipid raft-mediated internalization, and membrane fusion 13. Normally, more than one route is involved in sEV uptake, which is largely determined by the origin of the sEV and the type of recipient cell 30. To identify the mechanisms involved in BMSC-derived sEV internalization, MM cells were treated with a variety of endocytosis inhibitors, including heparin, cytochalasin D, dynasore, genistein, and chlorpromazine 13, 30. MM1S cells were treated with different concentrations of inhibitors and the uptake of DID-labeled BMSC-derived sEVs was evaluated. Heparin decreased of sEV uptake internalization to a similar degree (~50%) at 5, 10, or 20 µg/ml, while cytochalasin D reduced sEV uptake in a dose-dependent manner, reaching a 60% decrease at the highest concentration (2 µM, Figure 4A and S4A-B). Treatment with the highest concentration of dynasore (100 µM) inhibited sEV uptake by 55% (Figure 4A) and genistein dose-dependently decreased internalization as much as 80% at the highest concentration (200 µM). 5 µM Chlorpromazine, an inhibitor of clathrin-mediated endocytosis, decreased sEV internalization by up to 30% only, and the highest concentration (20 µM) did not induce a significantly greater decrease (Figure 4A).

Next, we evaluated the effects of different concentrations of these inhibitors on MM1S cell viability. The highest concentrations of dynasore and genistein significantly decreased their cell viability (Figure 4B), suggesting that the uptake inhibition from a high dose of these inhibitors may be the result of a decrease in cell viability. Thus, for each inhibitor, we selected the concentration that induced minimal cell injury whilst retaining a high inhibition rate of sEV uptake. At the selected concentrations, the five inhibitors did not decrease the cell viability of the other three MM cell lines (Figure 4C), which were then used for sEV uptake assays. In RPMI 8226 and U266 cells, these inhibitors except for chlorpromazine significantly reduced sEV uptake (Figure 4D-E and S4A-B). In H929 cells, cytochalasin D, dynasore and genistein inhibited sEV uptake, but heparin and chlorpromazine did not (Figure 4F and S4A-B). We next confirmed these results using sEVs isolated from BMSCs of another MM patient. These sEVs also could attenuate MM cell chemosensitivity to bortezomib, as measured using luminescent cell viability assay and Cell Counting Kit-8 (Figure S5A-B). Dynasore was also able to dose-dependently inhibit the uptake of these sEVs in all these four MM cell lines (Figure S5C). As targets of dynasore and genistein are important in clathrin- and caveolin-mediated endocytosis, our results indicate that these two pathways are involved in the internalization of BMSC-derived sEVs by MM cells, and that inhibitors of these processes are able to suppress sEV uptake.

### Macropinocytosis is involved in sEV uptake by MM cells

**Figure 5:**
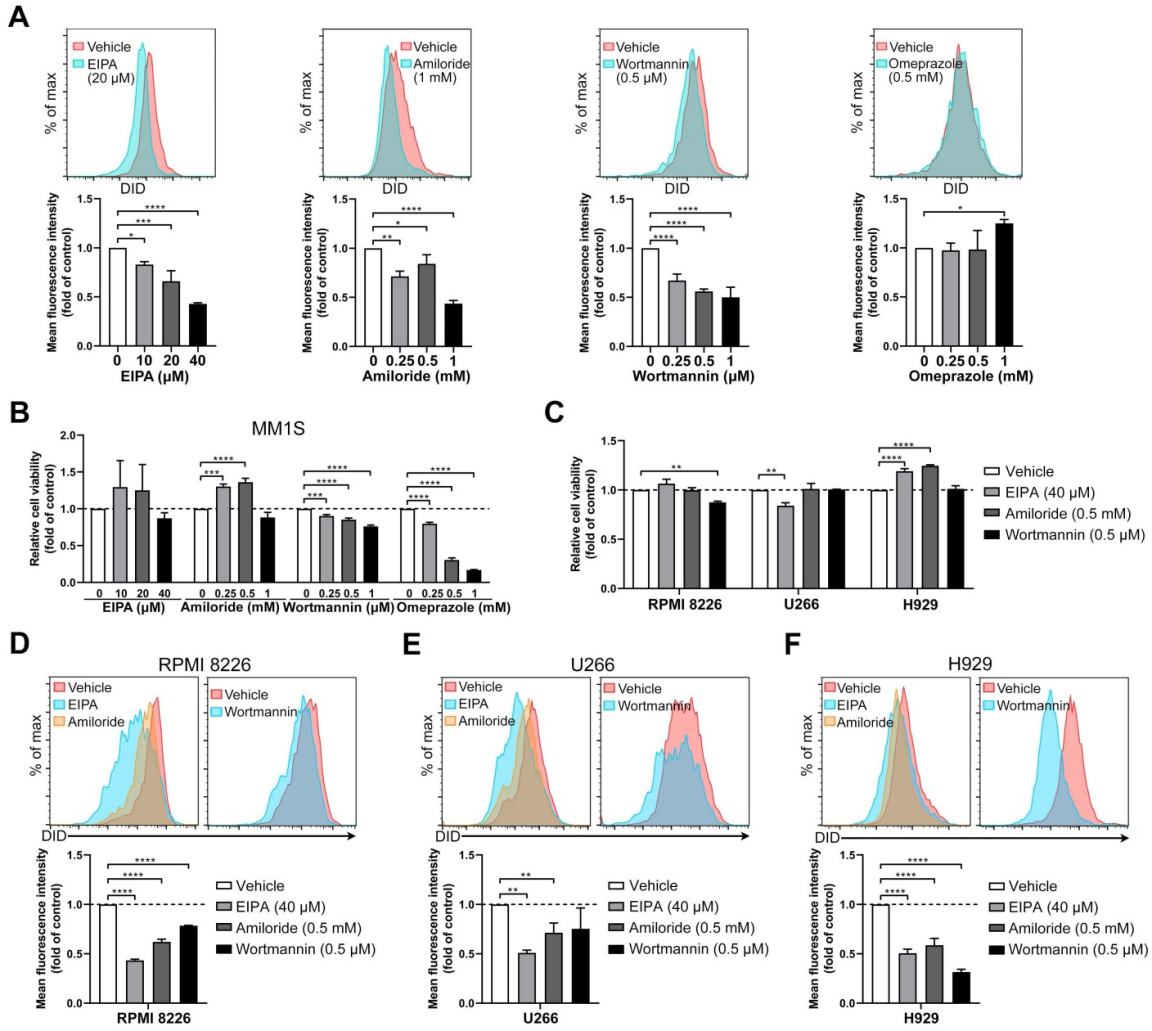
** Inhibitors of macropinocytosis impair the uptake of BMSC-derived sEVs by MM cells.** (A) MM1S cells pre-treated with EIPA, amiloride, wortmannin, or omeprazole at the indicated concentrations for 30 min were cultured with 50 μg/mL DID-labeled BMSC-derived sEVs for another 4 h. The mean fluorescence intensity of DID in these cells was determined using flow cytometry. (B) MM1S cells were treated with EIPA, amiloride, wortmannin, or omeprazole at the indicated concentrations for 4.5 h in the absence of sEVs and their cell viability was measured. (C) RPMI 8226, U266, or H929 cells were treated with EIPA, amiloride, or wortmannin at the indicated concentration for 4.5 h in the absence of sEVs and their cell viability was measured. (D) RPMI 8226, (E) U266, or (F) H929 cells were pre-treated with EIPA, amiloride, or wortmannin at the indicated concentration for 30 min and cultured with 50 μg/mL DID-labeled BMSC-derived sEVs for another 4 h. The mean fluorescence intensity of DID in these cells was determined using flow cytometry. One-way ANOVA followed by multiple compressions was used for comparing multiple groups. n=3. Error bar, mean ± SD. *P < 0.05, **P < 0.01, ***P < 0.001, ****P < 0.0001.

To determine whether macropinocytosis and membrane fusion are associated with sEV uptake, MM cells were treated with four different inhibitors (EIPA, amiloride, wortmannin, and omeprazole). EIPA inhibits macropinocytosis through by sodium/proton exchanger 31, and it was found to inhibit sEV uptake in the present study. EIPA significantly dose-dependently decreased sEV internalization by MM1S cells (Figure 5A and S4C). Amiloride, an inhibitor of macropinocytosis, decreased sEV uptake by up to 60% (Figure 5A), and wortmannin (another macropinocytosis blocker) 32 dose-dependently inhibited the uptake of sEV in MM1S cells (Figure 5A). Omeprazole, a proton pump inhibitor 33, did not impair sEV uptake in MM1S cells (Figure 5A). The effects of these four inhibitors on MM1S cell viability were also determined (Figure 5B). Since it induced a substantial decrease in cell viability and was unable to decrease sEV uptake, omeprazole was not used for further experimentation.

A safe and effective concentration of the remaining three inhibitors was selected, exhibiting mild cytotoxicity in RPMI 8226, H929 and U266 cells (Figure 5C). EIPA and amiloride reduced sEV uptake in RPMI 8226, H929 and U266 cells, while wortmannin did so in RPMI 8226 and H929, but not in U266 cells (Figure 5D-F and S4B-D). These results indicate a contribution from macropinocytosis to sEV uptake by MM cells and suggest that these four MM cell lines differ in the detailed mechanisms of sEV uptake since macropinocytosis inhibitors act differently in different MM cell lines.

### Endocytosis-associated proteins are involved in sEV internalization by MM cells

**Figure 6:**
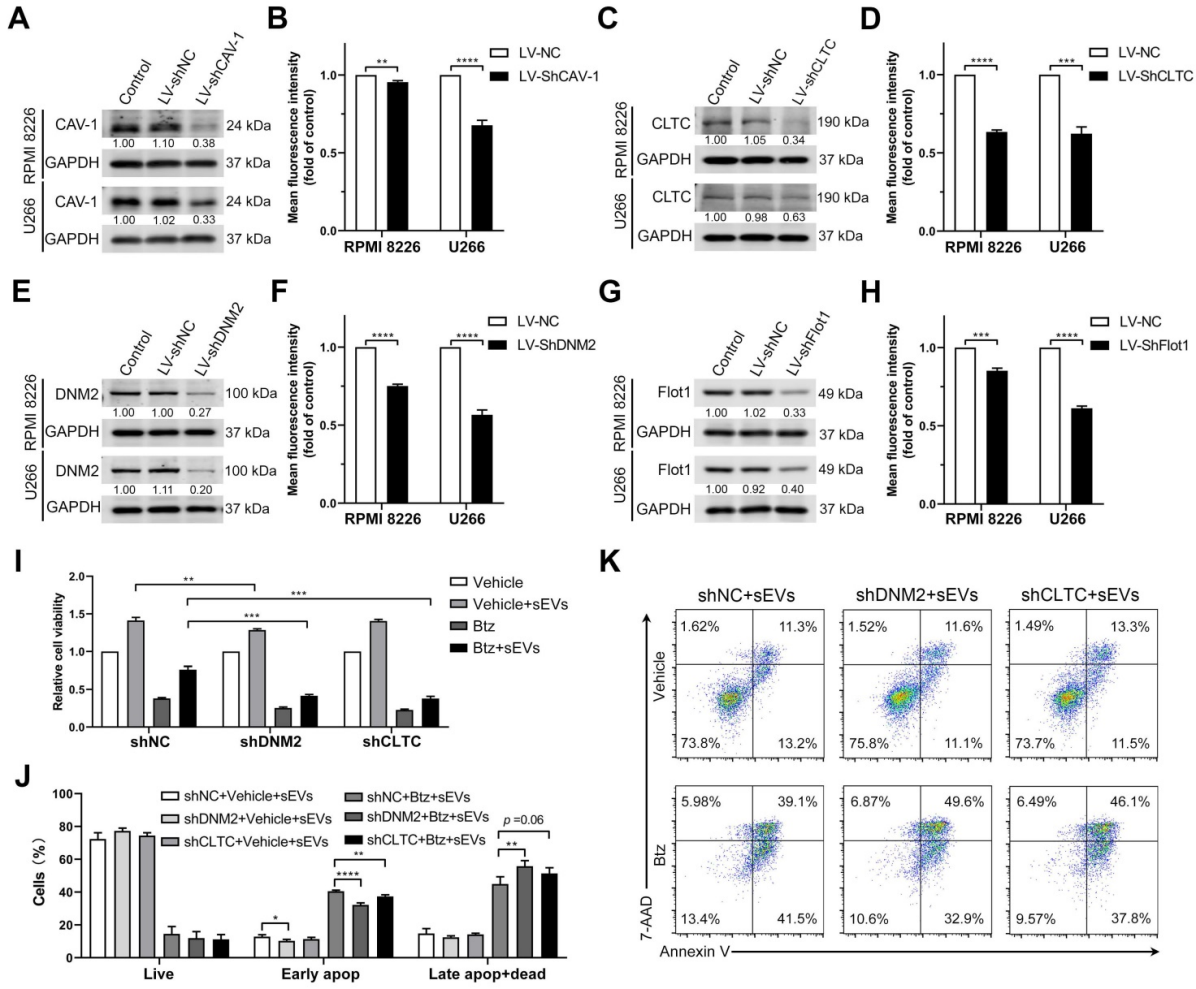
** Endocytosis-related proteins are involved in the uptake of BMSC-derived sEVs by MM cells.** RPMI 8226 and U266 cells were infected with lentivirus expressing shRNA against key endocytic proteins, including (A) CAV-1, (C) CLTC, (E) DNM2, and (G) Flot1 or negative control shRNA (shNC) and the expression of these proteins was determined using western blot. The pixel density of endocytosis-related proteins was quantified and normalized to GAPDH. RPMI 8226 and U266 cells infected with lentivirus expressing shRNA against key endocytic proteins, including (B) CAV-1, (D) CLTC, (F) DNM2, and (H) Flot1, or shNC were cultured with 50 μg/mL DID-labeled sEVs for 4 h and the mean fluorescence intensity of DID in these cells was determined using flow cytometry. Student's t-test was used for comparing two groups. (I-K) RPMI 8226 cells were infected with lentivirus expressing shRNA against DNM2 or CLTC or negative control shRNA (shNC) for 24 h and treated with or without bortezomib in the presence or absence of 100 μg/mL sEVs for another 48 h. (I) Their cell viability was measured using a luminescent cell viability assay. One representative result in triplicate of three experiments was presented by histograms. (J) Apoptotic cells were determined using 7-AAD and Annexin-V staining and flow cytometry. The proportions of live, early apoptotic or late apoptotic and dead cells were analyzed and presented by histograms. (K) Representative flow cytometry plots are shown. One-way ANOVA followed by multiple compressions was used for comparing multiple groups. Error bar, mean ± SD. **P < 0.01, ***P < 0.001, ****P < 0.0001.

As inhibitors of endocytosis significantly suppress sEV uptake, shRNA-mediated knockdown of endocytosis-associated proteins was used to explore the key contributors to these processes. These endocytic proteins included caveolin-1 (CAV-1), clathrin heavy chain (CLTC), dynamin-2 (DNM2), and Flot1, key modulatory proteins of different subclasses of clathrin- and caveolin-mediated endocytosis. Lentiviral shRNAs-knockdown induced a significant decrease in the expression of these target proteins in RPMI 8226 and U266 cells (Figure 6A, C, E and G) and has minor effect on their cell viability (Figure S6A). Knockdown of CAV-1 mildly inhibited sEV internalization in RPMI 8226 cells by up to 5% and significantly decreased this process by 30% in U266 cells (Figure 6B). Likewise, CLTC knockdown significantly decreased sEV uptake by an average of 40% in both RPMI 8226 and U266 cells (Figure 6D). DNM2 knockdown decreased sEV uptake by an average of 25% and 45% in RPMI 8226 and U266 cells, respectively (Figure 6F). Flot1 knockdown resulted in an average decrease of 20% and 40% sEV internalization in RPMI 8226 and U266 cells, respectively (Figure 6H). Next, the effect of DNM2 or CLTC knockdown on the chemosensitivity to bortezomib in MM cells was investigated. Both DNM2 and CLTC knockdown further decreased the cell viability after treatment with bortezomib in the presence of sEVs (Figure 6I). In the absence of sEVs, knockdown of DNM2 or CLTC did not induce further changes in the apoptosis of MM cells treat with or without bortezomib (Figure S6B), whereas they significantly decreased the proportion of early apoptotic cells after treatment with bortezomib in the presence of sEVs (Figure 6J-K). Additionally, knockdown of DNM2 also significantly increased the proportion of late apoptotic cells in the presence of bortezomib and sEVs, and CLTC knockdown induced the similar effect with a trend close to significance (Figure 6J-K). However, this effect faded away after a long-term culture of these DNM2 or CLTC knockdown cells (Figure S6C-D), suggesting that compensatory mechanisms may be involved in the recovery of uptake pathways after the gene knockdown. These results demonstrate that these endocytosis-related proteins contribute to BMSC-derived sEV uptake by MM cells, and further confirm that clathrin- and caveolin-mediated endocytosis are the primary pathways of sEV-mediated communication between BMSCs and MM cells.

### Dynasore inhibits sEV uptake in primary MM cells

**Figure 7:**
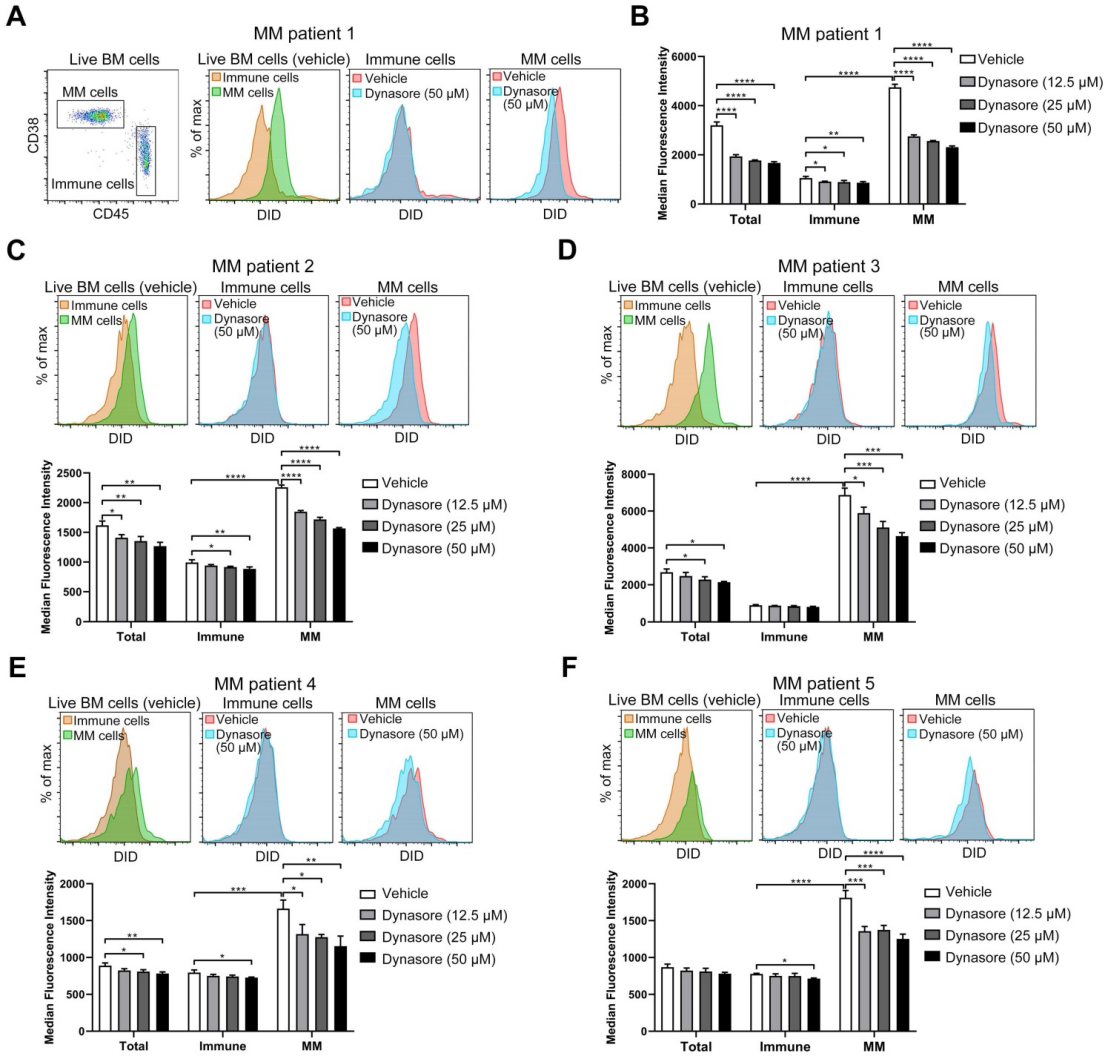
** Dynasore inhibits sEV uptake in primary MM cells.** (A-F) BMMCs obtained from 5 MM patients were pre-treated with or without dynasore at different final concentrations (12.5, 25, or 50 µM) for 30 min and then incubated with 50 μg/mL DID-labeled sEVs for another 4 h. After staining with anti-mouse CD45-PE, anti-mouse CD38-Pacific Blue, and Annexin V-FITC, cells were acquired using flow cytometry. Live BM cells (Annexin V^-^) were gated and analyzed. (A) Gating of MM (CD45^-^CD38^+^) and CD45^+^ immune cells from MM patient 1 (left panel). Representative flow cytometry plots showing the DID signal in MM patient 1 MM and immune cells treated with or without dynasore. (B) Median florescence intensity of DID in total, MM, and immune cells was analyzed and presented by histograms. Same analyses were performed using BMMCs isolated from MM patient (C) 2, (D) 3, (E) 4, and (F) 5. One-way ANOVA followed by multiple compressions was used for comparing multiple groups. Error bar, mean ± SD. *P < 0.05, **P < 0.01, ***P < 0.001, ****P < 0.0001.

Given that dynasore stably inhibited sEV uptake in four MM cell lines and that knockdown of its specific target DNM2 also reduced sEV uptake, we next determined the inhibitory effect of this inhibitor on sEV uptake in primary MM cells. BMMCs were isolated from the BM cells of MM patients and then cultured with DID-labeled sEVs in the presence of dynasore. As the loss of CD138 during cold storage and processing frequently occurs 34, 35, cells with a CD38^high^CD45^-/dim^ phenotype were defined as malignant MM cells and rest of CD45^+^ cells were considered as healthy immune cells (Figure 7A). Without dynasore treatment, DID signal in MM cells was stronger than that in immune cells after the co-culture of BMMCs with DID-labeled sEVs (Figure 7A-B). Similar result was observed in the other four BM samples from different MM patients (Figure 7C-F). These results suggested that MM cells always take up more sEVs than healthy immune cells. Dynasore always inhibited the uptake of sEVs by MM cells and healthy immune cells (Figure 7A-F). Moreover, 50 μM dynasore decreased sEV uptake by an average of 51% and 33% in MM cells obtained from MM patient 1 and 3, respectively, and by ~31% in the other 3 MM patients (Figure S7A-E). However, it reduced sEV uptake by 20% in average in healthy immune cells of patient 1 and by up to 10% in the other MM BM samples (Figure S7A-E), suggesting that dynasore-mediated inhibition of sEVs uptake in MM cells is stronger than that in healthy immune cells. These ex vivo results, showing dynasore preferentially suppressed the uptake of sEV by MM cells, imply that inhibitor of sEV uptake may serve as a potential strategy for improving MM treatment.

### Inhibiting endocytosis enhances MM cell chemosensitivity to bortezomib

**Figure 8:**
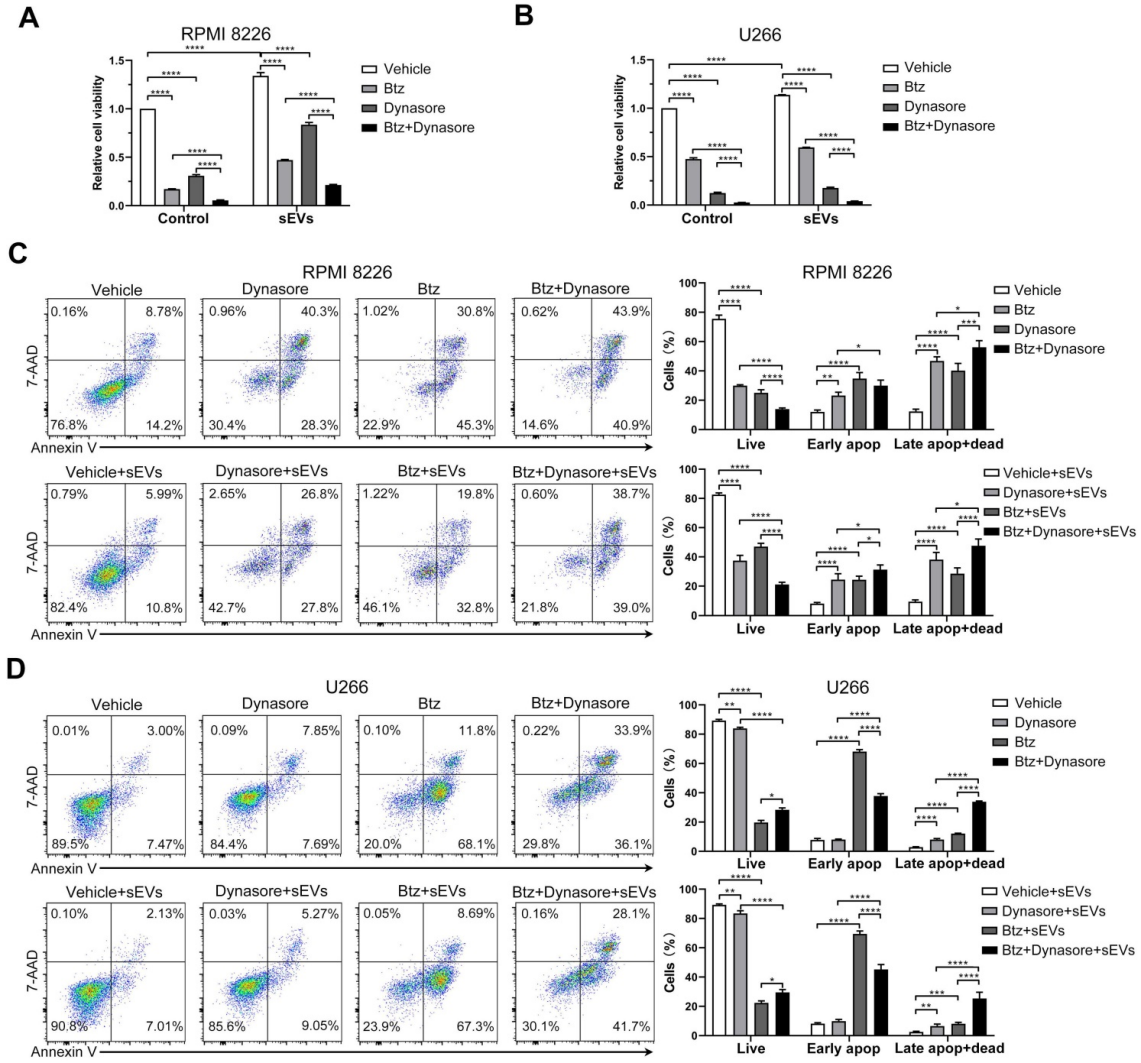
** Endocytosis inhibitor enhances the anti-MM effect of bortezomib in vitro.** (A) RPMI 8226 or (B) U266 cells were pretreated with or without bortezomib for 6 h and then cultured with or without dynasore in the presence or absence of 100 μg/mL sEVs for another 24 h, and the cell viability was measured. One representative result in triplicate of three experiments was presented by histograms. Similar results were obtained in three independent experiments. (C) RPMI 8226 or (D) U266 cells were pretreated with or without bortezomib for 6 h and then cultured with or without dynasore in the presence or absence of 100 μg/mL sEVs for another 24 h, apoptotic cells were determined using 7-AAD and Annexin-V staining and flow cytometry. Representative flow cytometry plots are shown. The proportions of live, early apoptotic or late apoptotic and dead cells were analyzed and presented by histograms. One-way ANOVA followed by multiple compressions was used for comparing multiple groups. Error bar, mean ± SD. *P < 0.05, **P < 0.01, ***P < 0.001, ****P < 0.0001.

Since BMSC-derived sEVs can reduce MM cell chemosensitivity, we next investigated whether blocking sEV uptake reversed bortezomib resistance in MM cells. To avoid the effects of dynasore on bortezomib-induced cytotoxicity, we first treated cells with bortezomib and then inhibited the sEVs uptake with dynasore. In the presence or absence of BMSC-derived sEVs, dynasore enhanced the bortezomib-induced decrease in both RPMI 8226 and U266 MM cells (Figure 8A-B). In the presence of bortezomib and sEVs, a further significant decrease in viable RPMI 8226 cells, and an increase percentage of apoptotic and dead cells, was observed following dynasore administration (Figure 8C). Conversely, in the presence or absence of sEVs, dynasore did not affect the bortezomib-induced decrease in viable U266 cells, but significantly increased bortezomib-induced late apoptosis and death (Figure 8 D). These in vitro data support that endocytosis inhibitor dynasore can enhance anti-MM effects of bortezomib.

### Dynasore enhances the anti-MM effect of bortezomib in vivo and prolongs the survival of MM mice

**Figure 9:**
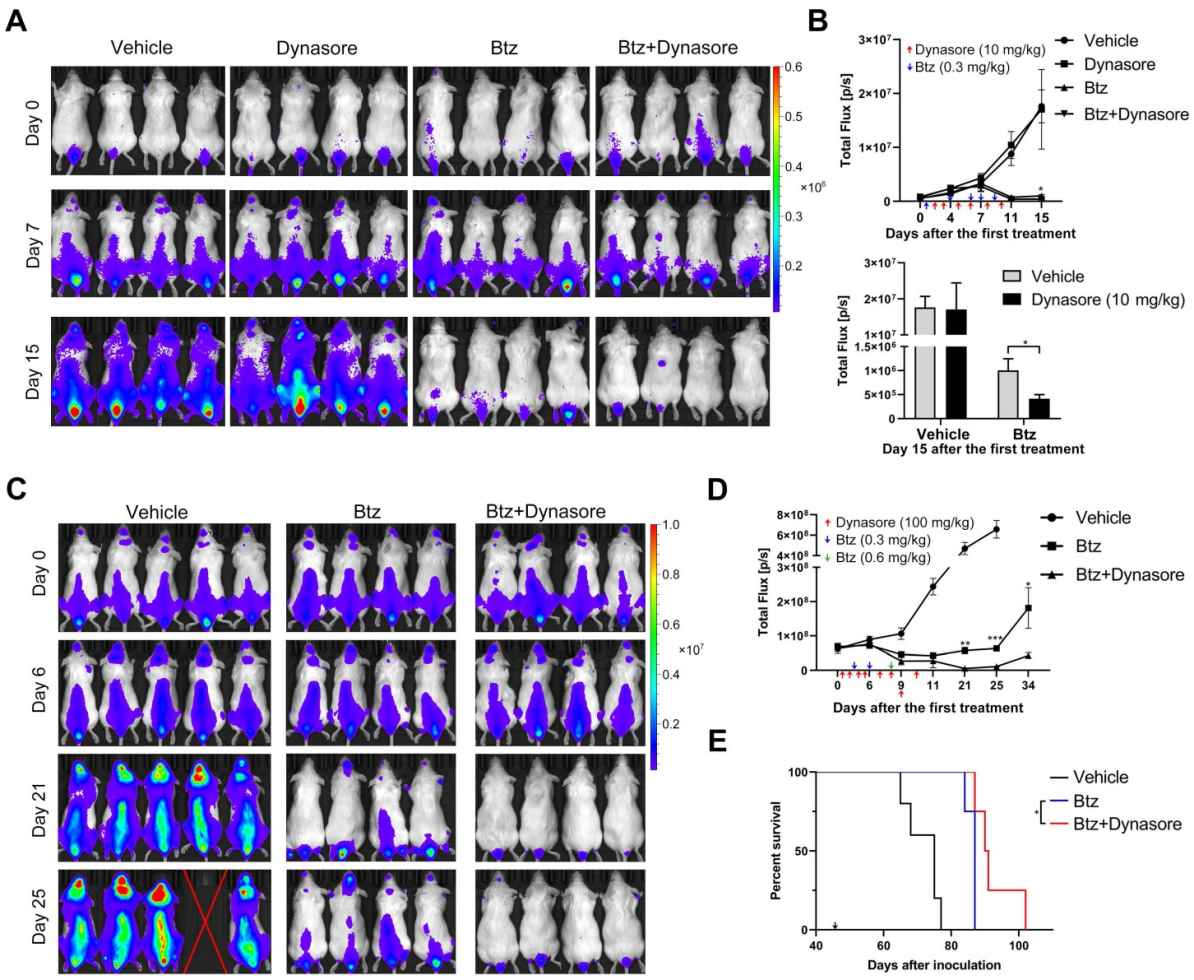
** Endocytosis inhibitor enhances the anti-MM effect of bortezomib in vivo and prolongs the survival of MM mice.** (A) B-NDG mice inoculated with U266-GFP-Luc cells were treated with bortezomib (Btz) with or without dynasore. At day 2, 3, 5, 6, 7, and 9 after the first treatment, 10 mg/kg dynasore was intraperitoneally injected. 0.3 mg/kg bortezomib were intraperitoneally injected at day 1, 4, 6, 7, 9 after the first treatment. The distribution of MM cells in these mice was measured at the indicated days after the first treatment using a living imaging system. 4 representative mice per group are shown. (B) The total flux in each mouse after treatment for the indicated days was determined and presented (upper panel). Arrows indicate the doses and time points of treatments. The total flux in mice of all groups at day 15 after the first treatment was presented by histogram (lower panel). n (vehicle) =8, n (dynasore) =5, n (btz or btz+dynasore) =6. (C) B-NDG mice inoculated with U266-GFP-Luc cells were treated with bortezomib (Btz) with or without dynasore. At day 1, 2, 4, 5, 7, 8, 9, and 10 after the first treatment, 100 mg/kg dynasore were intraperitoneally injected. 0.3 mg/kg bortezomib were intraperitoneally injected at day 3 and 6 after the first treatment, and at day 8, 0.6 mg/kg bortezomib were intraperitoneally injected. The distribution of U266-GFP-Luc cells in these mice was measured at the indicated days after the first treatment using a living imaging system. (D) The total flux in each mouse after treatment for the indicated days was determined. Arrows indicate the doses and time points of treatments. n (vehicle) =5, n (btz or btz+dynasore) =4, Error bar, mean ± SEM. Student's t-test was used for comparing two groups. (E) Moribund mice with hindlimb paralysis were sacrificed and the Kaplan-Meier curve was established. Arrow indicates the day of the first treatment. *P < 0.05.

Next, we studied the* in vivo* effect of dynasore on MM growth using a xenograft MM mouse model. Dynasore was injected daily to maximize its effect on sEV uptake *in vivo*. Injection of 10 mg/kg dynasore alone did not induce a decrease of MM burden, whereas it further reduced the decrease of MM burden caused by bortezomib (Figure 9A-B). However, 10 mg/kg dynasore-induced enhancement of MM inhibition was mild and thus its dose was raised to 100 mg/kg. Compared with bortezomib alone, co-treatment with 100 mg/kg dynasore and bortezomib further inhibited U266 cell xenograft growth *in vivo* after 21 days of the first treatment (Figure 9C-D). 100mg/kg dynasore alone did not induce a reduction of MM burden *in vivo* during the injection period, whereas 11 days after dynasore withdrawal, it reduced the mean level of MM without statistical significant (Figure S8A-B), suggesting a possible late effect of dynasore treatment on MM inhibition. Moreover, compared to bortezomib, injection of 100mg/kg dynasore significantly prolonged the survival of MM mice (Figure 9E), demonstrating a complementary role of dynasore in bortezomib-based MM therapy.

## Discussion

Compelling evidence demonstrates that EVs facilitate communication between cells and regulators of the tumor microenvironment 6, 36. The sEV-mediated interplay between BMSCs and MM cells directly promotes MM progression and drug resistance. By enhancing the angiogenesis and immunosuppression, sEVs also facilitate the establishment of a BM microenvironment appropriate for the progression of MM 8, 27, 37. Various routes and mechanisms, including membrane fusion, clathrin- or caveolin- dependent endocytosis, phagocytosis, and macropinocytosis, have been reported to be involved in EV internalization 13, 14. However, these uptake mechanisms are not consistent, and are largely determined by the cellular origin of the EV and recipient cell type. Considering the necessary involvement of BMSC-derived sEVs in MM progression, elucidating the detailed cellular mechanisms involved in sEV internalization may encourage the development of alternative anti-MM strategies, which work by blocking sEVs-mediated cellular communications. In the present study, we revealed that BMSC-derived sEVs predominantly enter MM cells via clathrin- or caveolin- dependent endocytosis and macropinocytosis. Moreover, blocking the endocytic pathway attenuates sEV-induced reduction of chemosensitivity to bortezomib, and enhances the anti-MM effect of bortezomib *in vivo*. These data indicate that blocking sEV internalization may be a promising strategy for the MM treatment.

In the present study, the contribution of human BMSC-derived sEVs to cell cycle regulation and chemosensitivity to bortezomib was first confirmed in four human MM cell lines. sEVs derived from mesenchymal stromal cells have been shown to upregulate the expression of cell-cycle-associated proteins such as cyclin D1 and cyclin E, and thus induce quiescent hepatocytes to re-enter the cell cycle 38. Furthermore, human umbilical cord mesenchymal stromal cell-derived sEVs are able to accelerate the proliferation of vaginal epithelial cells by promoting mitosis 39. These findings are consistent with those of the present study, where an increase mitotic MM cells was observed following exposure to BMSC-derived sEVs. Our data also indicate elevated sEV uptake capacity in MM cells in the S and G_2_/M phases, suggesting that dividing cells internalize sEVs more readily than quiescent cells, and that sEVs subsequently promote further mitosis and cellular proliferation.

Chemical inhibitors that block specific uptake pathways are frequently used to assess the mechanisms of EV uptake under different conditions and in various cell types 13. Heparin sulphate proteoglycans (HSPGs) favor the entry of viral particles and lipoproteins, and treatment with the soluble analogue heparin reduces the uptake of sEVs released from bladder cancer 40, human glioblastoma 41 and epidermoid carcinoma cells 30. Our results revealed a ~50% decrease in sEV uptake following heparin treatment, further confirming the involvement of HSPGs in MM cell sEV internalization. Cytochalasin D, a metabolite known to inhibit actin polymerization, has been reported to significantly reduce EV uptake in various cell types 30, 42. It also significantly inhibits the uptake of sEVs by MM cells, suggesting that this process requires cytoskeletal remodeling. The tyrosine kinase inhibitor genistein is able to reduce simian virus 40 internalization and sEV uptake by disrupting the actin network and inhibiting the recruitment of dynamin to the cell membrane 13, 43. Since the targets of genistein are implicated in clathrin-independent endocytosis 43, the genistein-induced inhibition of sEV uptake by MM cells suggests a role for clathrin-independent endocytosis in the internalization of BMSC-derived sEVs. Chlorpromazine targets several cellular membrane molecules, including receptors of dopamine, serotonin, muscarinic acetylcholine, and histamine, and thus disrupts the formation of clathrin-coated pits 44. Chlorpromazine-induced inhibition of sEV uptake has been reported in a number of adherent cell types, such as ovarian cancer cells, stromal cells, and macrophages 14, 31, 45. In the present study, a low concentration of chlorpromazine exerted a mild decrease in sEV uptake in only one MM cell line, indicating that the uptake of BMSC-derived sEVs by suspension MM cells may not rely on chlorpromazine-targeted receptors.

Both EIPA and amiloride are inhibitors of sodium/proton exchange and are known to reduce submembranous pH, resulting in decreased actin assembly and macropinocytosis 42, 46. Wortmannin is an inhibitor of phosphoinositide 3-kinase which is important for the initiation of membrane ruffling and macropinosomes formation during macropinocytosis 32. These three inhibitors prevented the internalization of BMSC-derived sEVs by MM cells, thus suggesting the involvement of macropinocytosis. Omeprazole is a proton pump inhibitor which suppresses EV-to-cell membrane fusion 33. Our results suggested that omeprazole does not inhibit the uptake of BMSC-derived sEVs and significantly impairs MM cell viability, suggesting that membrane fusion may not be a primary route of sEV uptake in MM cells.

DNM2 is a GTPase and its accumulation in nascent clathrin-coated pits is required for membrane fission and clathrin-coated vesicle release 47. DNM2 also facilitates the assembly and expansion of caveolar vesicles during both clathrin- and caveolin-mediated endocytosis 48, 49. Retarding DNM2 with chemical inhibitors markedly reduces the internalization of sEVs by macrophages, microglia, and stromal cells 14, 42, 50. Dynasore mainly inhibits the GTPase activity of dynamin-1 (DNM1), DNM2, and the mitochondrial dynamin Drp1, but not of other small GTPases 51. DNM1 is predominantly expressed in neuronal tissue 52, 53, whereas DNM2 is expressed ubiquitously throughout the body 54, 55. Thus, dynasore acts as a potent inhibitor of endocytic pathways mainly through targeting DNM2 in MM cells. In the present study, both chemical inhibition and shRNA-mediated knockdown of DNM2 induced a significant decrease in BMSC-derived sEV uptake in MM cells, indicating that DNM2 mediates sEV uptake. Moreover, both CAV-1 and CLTC knockdown resulted in decreased sEV uptake, further highlighting the important roles of clathrin- and caveolin- mediated endocytosis in BMSC-derived sEV internalization.

Since sEV-mediated communication facilitates tumor progression via multiple different pathways, blocking sEV secretion, eradicating them from the circulation, or preventing sEV uptake may be beneficial during tumor treatment. Inhibitors targeting the release of EVs, such as GW4869, spiroepoxide, and manumycin-A, have been reported to disrupt EV-mediated communication and thus support cancer treatment *in vitro* and *in vivo* 56-59. Based on our results, we used dynasore to block sEV uptake, substantially enhancing the anti-MM effect of bortezomib *in vitro* and in a human MM xenograft mouse model. Interestingly, MM cells always take up more sEVs than healthy immune cells *ex vivo* and dynasore-mediated inhibition of sEV uptake in MM cells is always stronger than healthy immune cells. This result can be attributed to the fact that malignant MM cells need to take up more EVs than healthy cells to promote their rapid proliferation, resulting in this process to be more easily suppressed by endocytosis inhibitors. These ex vivo results, together with the evidence in the mouse model, further imply that blocking sEV-mediated communication may serve as a novel therapeutic strategy for MM treatment.

In conclusion, the results of the present study confirm the importance of human BMSC-derived sEVs in MM cell's chemosensitivity to bortezomib and provide insights to improve our understanding of the molecular mechanisms by which these cells internalize BMSC-derived sEVs. Our results not only suggest that clathrin- and caveolin-dependent endocytosis and macropinocytosis are predominant endocytic routes of sEV uptake, but also demonstrated that inhibiting sEV endocytosis may be a novel and potent strategy for improved MM treatment. However, further research is required to enhance the specificity and efficiency of sEV uptake blockade, and thus further improve their anti-MM efficacy.

## Supplementary Material

Supplementary method, figures and table.

## Abbreviations

7-AAD: 7-Aminoactinomycin D
BM: bone marrow
BMMC: bone marrow mononuclear cells
BMSC: bone marrow stromal cell
CAV-1: caveolin-1
CLTC: clathrin heavy chain
DNM2: dynamin-2
EIPA: 5-(N-Ethyl-Nisopropyl) amiloride
EVs: extracellular vesicles
FBS: fetal bovine serum
Flot1: flotillin-1
GAPDH: glyceraldehyde-3-phosphate dehydrogenase
HSPGs: heparin sulphate proteoglycans
MDSC: myeloid-derived suppressor cell
MM: multiple myeloma
PBS: phosphate buffer saline
sEVs: small extracellular vesicles
TEM: Transmission electron microscopy
