# Supplementary material for: Endocytic pathway inhibition attenuates extracellular vesicle-induced reduction of chemosensitivity to bortezomib in multiple myeloma cells
Source: Theranostics. 2021 Jan 1;11(5):2364–80. doi: 10.7150/thno.47996 (PMC7797667; doi:10.7150/thno.47996)
Supplement: Supplementary file 1 — Supplementary method, figures and table. [file thnov11p2364s1.pdf]

Supplemental material

Supplemental method

TUNEL apoptosis assay

Bone marrow stromal cells (BMSCs) were cultured in serum-free medium for 24 h and then fixed in 4% paraformaldehyde for 30 min. After washing with phosphate buffer saline (PBS), these cells were incubated with PBS containing 0.3% Triton X-100 for 5 min. These cells were washed with PBS and then incubated with TUNEL staining buffer (Beyotime Biotechnology, Shanghai, China) containing terminal deoxynucleotidyl transferase and Cy3-labeled dUTP for 60 min. After washing, these BMSCs were stained with DAPI and images of cells were acquired with the Leica SP8 confocal microscope (Leica, Wetzlar, Germany).

Supplemental Figures

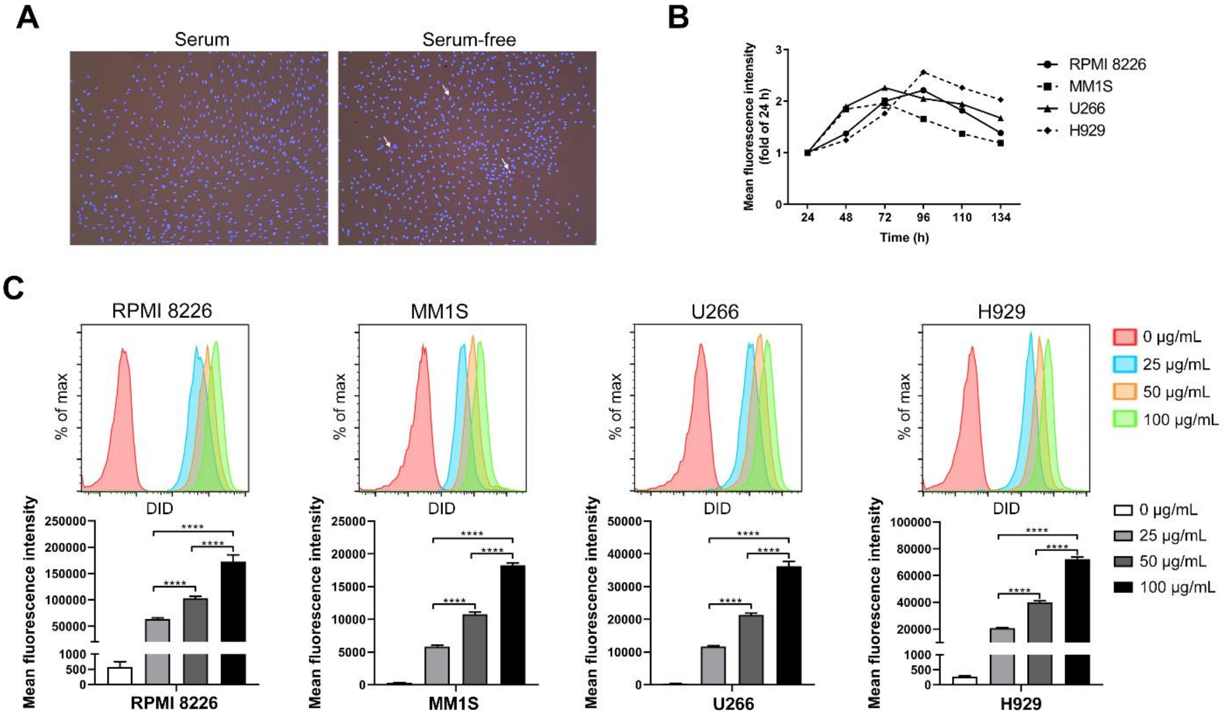

Figure S1. (A) BMSCs were cultured in serum-free medium for 24 h and then stained with a TUNEL apoptosis detection kit. (B) 50 μg/mL DID-labeled BMSC-derived sEVs were added to four MM cell lines, including RPMI 8226, MM1S, U266, and H929, and the mean fluorescence intensity of DID in these cells was determined using flow cytometry after the culture for indicated times. (C) RPMI 8226, MM1S, U266, and

H929 cells were cultured with or without 25, 50, or 100 μg/mL DID-labeled BMSC-derived sEVs for 24 h and the mean fluorescence intensity of DID in these cells was determined using flow cytometry. Original values of one representative were presented by histograms. ****P < 0.0001.

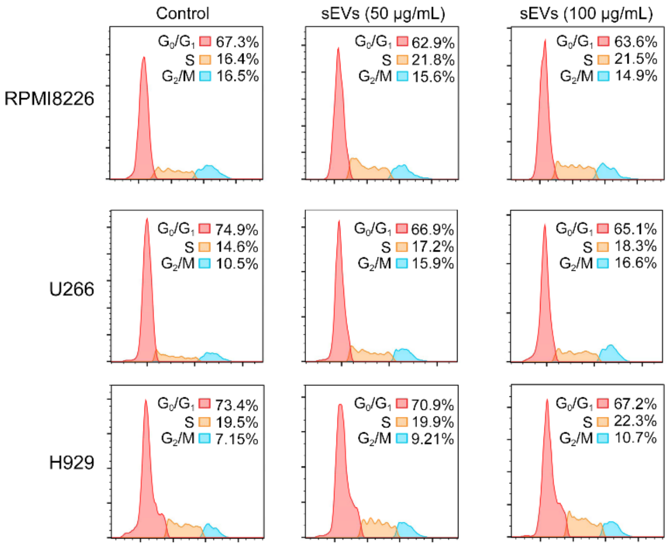

Figure S2. RPMI 8226, U266, and H929 cells were cultured with or without 50 or 100 μg/mL BMSC-derived sEVs for 48 h and the cell cycle was determined using PI staining and flow cytometry. Representative flow cytometry plots are shown.

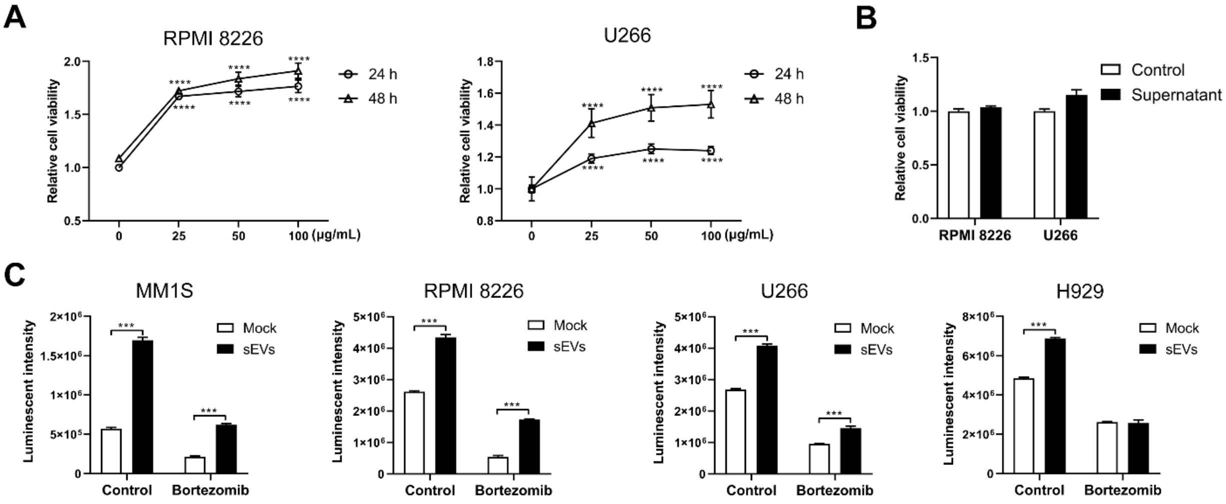

Figure S3. (A) RPMI 8226 or U266 were cultured with BMSC-derived sEVs at the indicated final concentrations for 24 or 48 h in serum-free medium. Relative cell viability was measured by a luminescent assay. (B) RPMI 8226 or U266 were cultured in medium without serum and treated with PBS (control) or supernatant without sEVs, for 48 h, and the cell viability was measured by a luminescent viability assay. (C)

MM1S, RPMI 8226, U266, or H929 cells were cultured with or without 100 μg/mL BMSC-derived sEVs in the absence or presence of bortezomib for 48 h and cell viability was measured by a luminescent assay. Original values of one representative were presented by histograms. **P* < 0.05, ***P* < 0.01, ****P* < 0.001, *****P* < 0.0001.

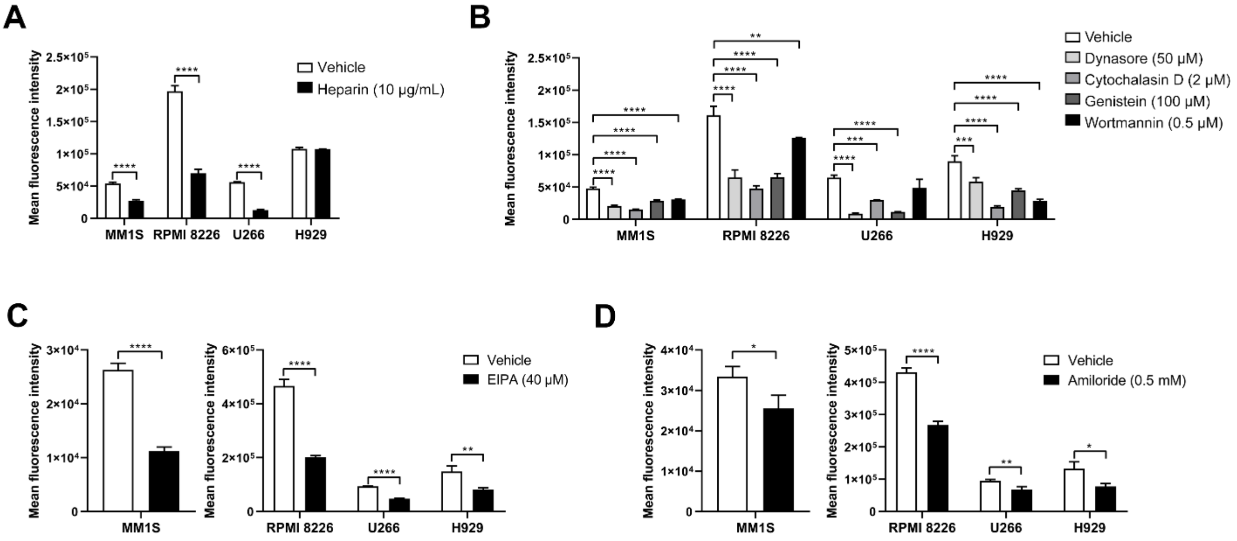

Figure S4. (A) MM1S, RPMI 8226, U266, or H929 cells were pre-treated with heparin at the indicated concentrations for 30 min and cultured with 50 μg/mL DID-labeled BMSC-derived sEVs for another 4 h. The mean fluorescence intensity of DID in these cells was determined using flow cytometry. (B) MM1S, RPMI 8226, U266, or H929 cells were pre-treated with dynasore, cytochalasin D, genistein, or wortmannin at the indicated concentrations for 30 min and cultured with 50 μg/mL DID-labeled BMSC-derived sEVs for another 4 h. The mean fluorescence intensity of DID in these cells was determined using flow cytometry. (C and D) MM1S, RPMI 8226, U266, or H929 cells were pre-treated with (C) EIPA or (D) amiloride, at the indicated concentrations for 30 min and then cultured with 50 μg/mL DID-labeled BMSC-derived sEVs for another 4 h. The mean fluorescence intensity of DID in these cells was determined using flow cytometry. Original values of one representative in triplicate were presented by histograms. **P* < 0.05, ***P* < 0.01, ****P* < 0.001, *****P* < 0.0001.

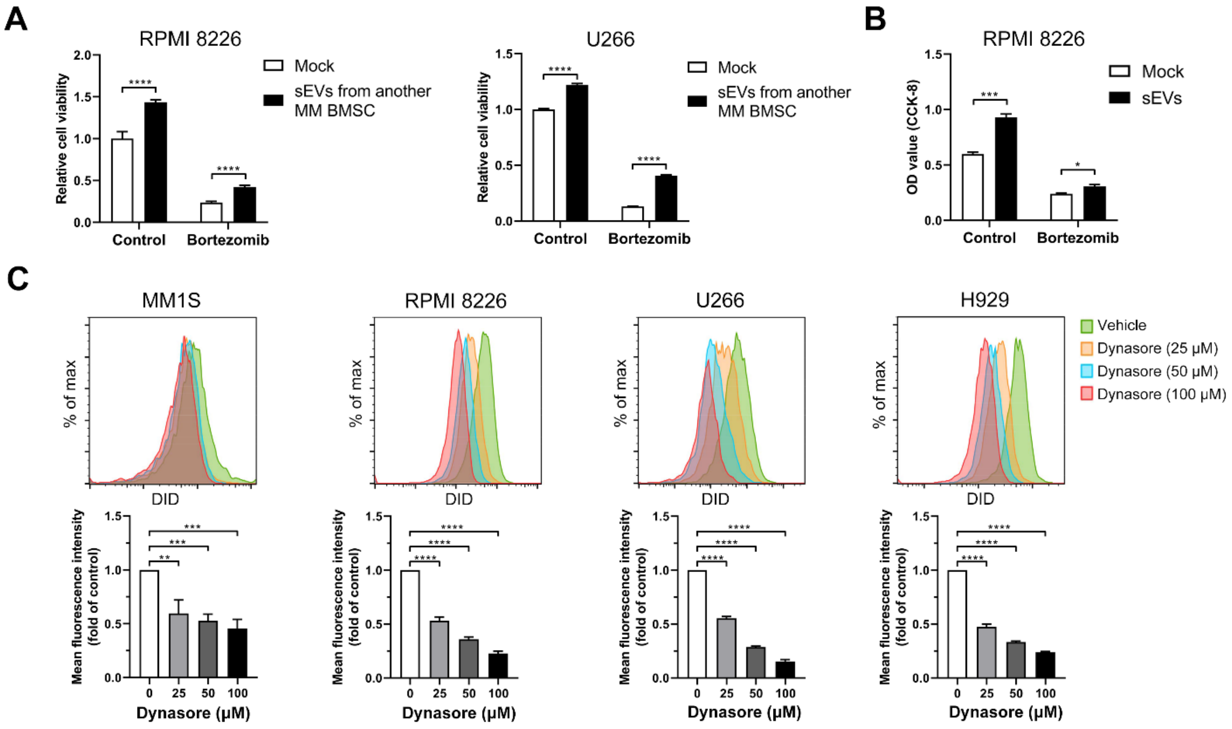

Figure S5. (A) RPMI 8226 or U266 cells were cultured with or without 100 μg/mL sEVs derived from another MM BMSC in the absence or presence of bortezomib for 48 h and cell viability was measured by a luminescent assay. (B). RPMI 8226 cells were cultured with or without 100 μg/mL sEVs derived from another MM BMSC in the absence or presence of bortezomib for 48 h and cell viability was measured using Cell Counting Kit-8 (CCK-8) assay. One representative result of three experiments was presented by histograms. Similar results were obtained in three independent experiments. (C) MM1S, RPMI 8226, U266, or H929 cells were pre-treated with dynasore at the indicated concentrations for 30 min and then cultured with 50 μg/mL DID-labeled sEVs derived from another MM BMSC for another 4 h. The mean fluorescence intensity of DID in these cells was determined using flow cytometry and presented by histograms. $^{**}P < 0.01$, $^{***}P < 0.001$, $^{****}P < 0.0001$.

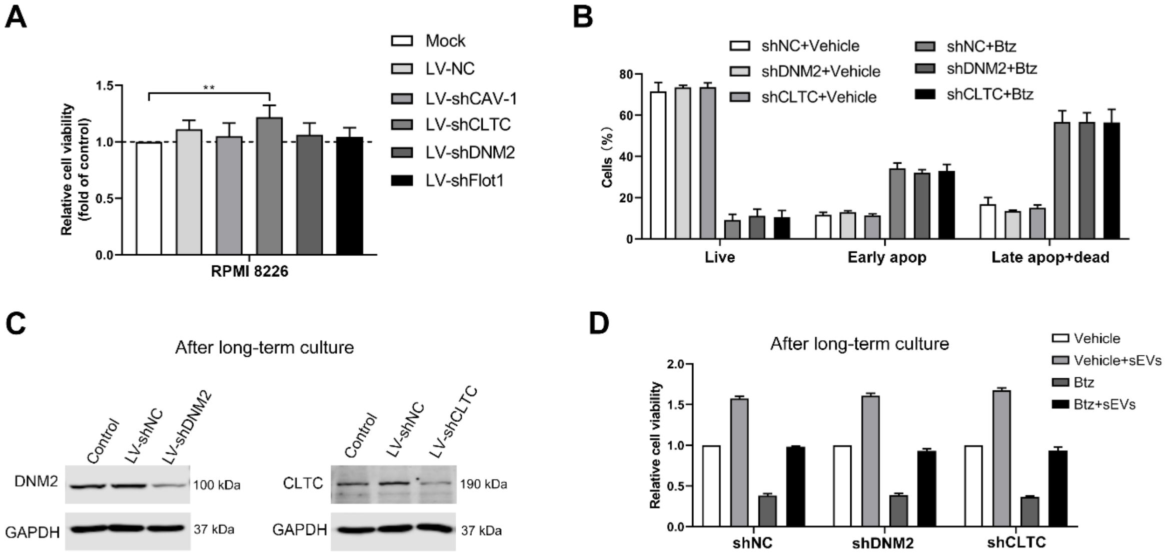

Figure S6. (A) RPMI 8226 cells were infected with lentivirus expressing shRNA against key endocytic proteins, including CAV-1, CLTC, DNM2, and Flot1 or negative control shRNA (shNC) for 48 h, and the cell viability was measured by a luminescent assay. (B) RPMI 8226 cells were infected with lentivirus expressing shRNA against DNM2 or CLTC or negative control shRNA (shNC) for 24 h and treated with or without bortezomib in the absence of 100 μg/mL sEVs for another 48 h. Apoptotic cells were determined using 7-AAD and Annexin-V staining and flow cytometry. The proportions of live, early apoptotic or late apoptotic and dead cells were analyzed and presented using histograms. (C) RPMI 8226 cells were infected with lentivirus expressing shRNA against DNM2 or CLTC or negative control shRNA (shNC) and cultured for more than one month in the presence of 1 μg/mL puromycin. The expression of DNM2 or CLTC in these cells was determined using western blot. (D) RPMI 8226 cells were infected with lentivirus expressing shRNA against DNM2 or CLTC or negative control shRNA (shNC) and cultured for more than one month in the presence of 1 μg/mL puromycin. These cells were treated with or without bortezomib in the presence or absence of 100 μg/mL sEVs for another 48 h and their cell viability was measured using a luminescent cell viability assay. ***P* < 0.01.

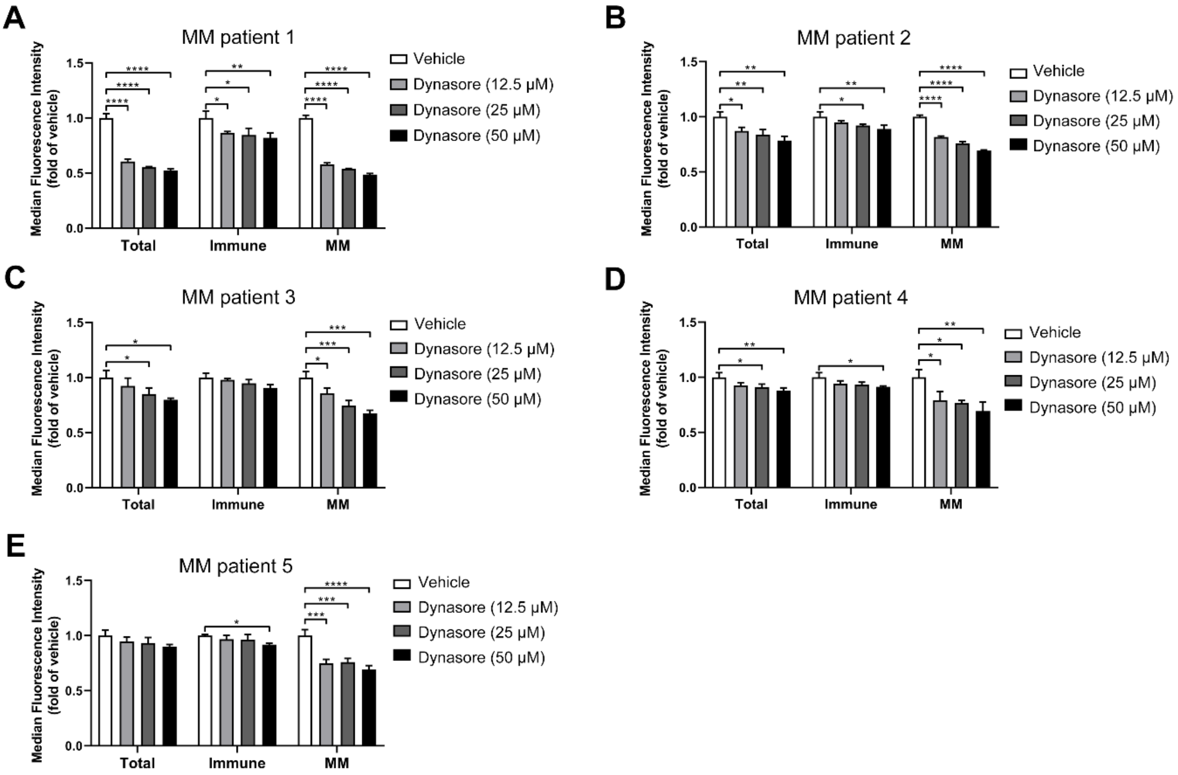

Figure S7. (A-E) Bone marrow mononuclear cells (BMMCs) obtained from 5 MM patients were pre-treated with or without dynasore at different final concentrations (12.5, 25, or 50 μM) for 30 min and then incubated with 50 μg/mL DID-labeled sEVs for another 4 h. The mean fluorescence intensity of DID in total, MM, or immune cells was determined using flow cytometry and presented as relative value by histograms. *P < 0.05, **P < 0.01, ***P < 0.001, ****P < 0.0001.

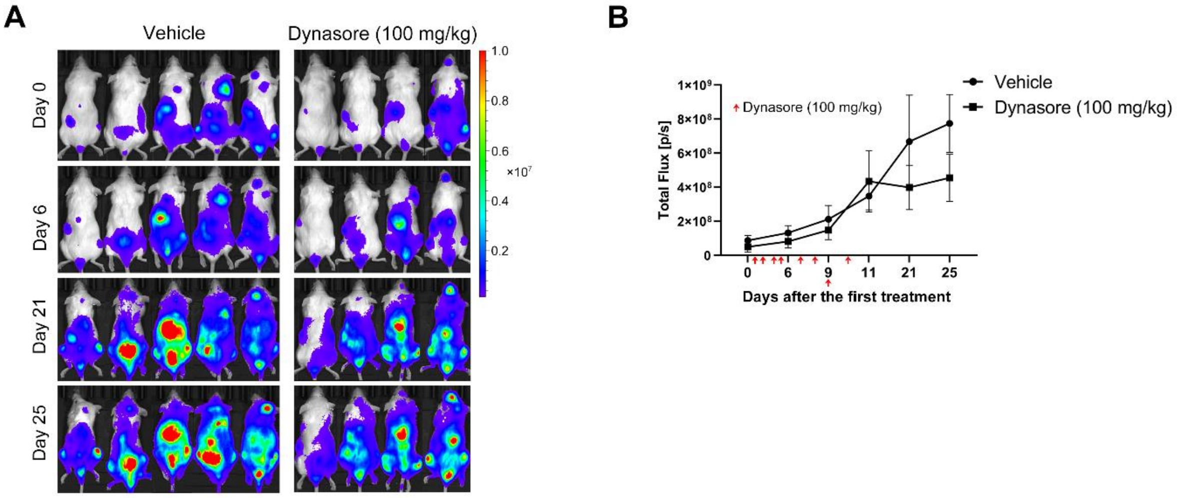

Figure S8. (A) B-NDG mice inoculated with MM-luc cells were treated with or without dynasore. At day 1, 2, 4, 5, 7, 8, 9, and 10 after the first treatment, 100 mg/kg dynasore was intraperitoneally injected. The distribution of MM cells in these mice was measured at the indicated days after the first treatment using a living imaging system. (B) The total flux in each mouse after treatment for the indicated days was determined. Arrows indicate the doses and time points of treatments. Error bar, mean ± SEM. n (vehicle) =5, n (dynasore) =4.

Supplemental tables

Table S1. Information of MM patients for ex vivo experiments

No.	Age	Sex	Disease	Therapy	MM load (% of BMMCs)
1	50	Female	MM	No*	60.4%
2	85	Male	MM	No*	50.5%
3	51	Female	MM	No*	29.7%
4	58	Male	MM	No*	13.3%
5	85	Male	MM	No*	9.72%

*No chemotherapy was received before sample collection. All these patients were newly diagnosed with MM. BMMCs, bone marrow mononuclear cells.
